# Intravital Imaging of Neocortical Heterotopia Reveals Aberrant Axonal Pathfinding and Myelination around Ectopic Neurons

**DOI:** 10.1093/cercor/bhab090

**Published:** 2021-04-20

**Authors:** Alice M Li, Robert A Hill, Jaime Grutzendler

**Affiliations:** Interdepartmental Neuroscience Program, Yale School of Medicine, New Haven, CT 06510, USA; Department of Neurology, Yale School of Medicine, New Haven, CT 06510, USA; Department of Pathology, Brigham and Women’s Hospital and Harvard Medical School, Boston, MA 02115, USA; Department of Neurology, Yale School of Medicine, New Haven, CT 06510, USA; Department of Biological Sciences, Dartmouth College, Hanover, NH 03755, USA; Interdepartmental Neuroscience Program, Yale School of Medicine, New Haven, CT 06510, USA; Department of Neurology, Yale School of Medicine, New Haven, CT 06510, USA; Department of Neuroscience, Yale School of Medicine, New Haven, CT 06510, USA

**Keywords:** axon pathfinding, cortical development, cortical malformation, heterotopia, myelination

## Abstract

Neocortical heterotopia consist of ectopic neuronal clusters that are frequently found in individuals with cognitive disability and epilepsy. However, their pathogenesis remains poorly understood due in part to a lack of tractable animal models. We have developed an inducible model of focal cortical heterotopia that enables their precise spatiotemporal control and high-resolution optical imaging in live mice. Here, we report that heterotopia are associated with striking patterns of circumferentially projecting axons and increased myelination around neuronal clusters. Despite their aberrant axonal patterns, *in vivo* calcium imaging revealed that heterotopic neurons remain functionally connected to other brain regions, highlighting their potential to influence global neural networks. These aberrant patterns only form when heterotopia are induced during a critical embryonic temporal window, but not in early postnatal development. Our model provides a new way to investigate heterotopia formation *in vivo* and reveals features suggesting the existence of developmentally modulated, neuron-derived axon guidance and myelination factors.

## Introduction

Up to one-third of routine postmortem examinations reveal the presence of neocortical heterotopia (Schulze and Braak 1978; Kaufmann and Galaburda 1989; Meencke and Veith 1992; Kasper et al. 1999), a heterogeneous group of focal cortical migration defects characterized by abnormally positioned clusters of neurons (Barkovich et al. 1992; Sisodiya 2004). Heterotopia have been linked to many neurological conditions including epilepsy, intellectual disability, and dyslexia (Galaburda and Kemper 1979; Hardiman et al. 1988; Palmini et al. 1991; Farrell et al. 1992; Meencke and Veith 1992; Nordborg et al. 1999; Kakita et al. 2002). However, comprehensive exploration of their pathogenesis and pathophysiology has been limited by a lack of tools for their investigation in the live animal. Several genetic, traumatic, chemotoxic, and other heterotopia models have been described (Riggs et al. 1956; Dvořàk et al. 1978; Sherman et al. 1987; Rosen et al. 1992; Ferrer et al. 1993; Amano et al. 1996; Brunstrom et al. 1997; Lee et al. 1997; Bai et al. 2003). However, these previous models have not been utilized for cellular intravital optical imaging analyses largely due to the lack of control over the position and timing of heterotopia induction and/or limited means for targeted cell labeling.

Here, we developed a methodology enabling the specific induction and visualization of focal heterotopia in the live mammalian neocortex. Our model uses *in utero* electroporation (Saito and Nakatsuji 2001; Tabata and Nakajima 2001; Lo Turco et al. 2009) combined with *in vivo* optical imaging, to generate and track layer I heterotopia, a poorly understood developmental malformation characterized by misplaced neurons localized near the pial surface (Jacob 1940; Morel and Wildi 1952; Schulze and Braak 1978; Galaburda et al. 1985; Kaufmann and Galaburda 1989; Yamamoto et al. 1997; Barkovich 1998). When visualized using label-free myelin imaging (Hill and Grutzendler 2014; Schain et al. 2014; Hill et al. 2018), we identify striking patterns of aberrantly projecting axons and focally increased myelination surrounding the heterotopic neurons. These distinct patterns emerge only when the heterotopia are induced during a critical embryonic period, suggesting the presence of locally derived, developmentally modulated signals that initiate the abnormal structural organization and myelination of the heterotopic axons. Identification and characterization of the neuronal and glial subtypes within embryonically induced heterotopia revealed many consistent features that are used to define spontaneously occurring heterotopia in both humans (Jacob 1940; Morel and Wildi 1952; Schulze and Braak 1978; Galaburda et al. 1985; Kaufmann and Galaburda 1989) and rodent models (Sherman et al. 1985, 1987, 1990; Ramos et al. 2008, 2014; Lipoff et al. 2011; Toia et al. 2017). Finally, using genetically encoded calcium biosensors, we demonstrate that heterotopic neurons display similar calcium transient frequencies as neighboring layer II/III neurons and respond to sensorimotor stimulation in behaving mice, opening new possibilities for the exploration of their influence on cortical function.

## Materials and Methods

### Animals

All experimental approaches and procedures were conducted in accordance with Yale University Institutional Animal Care and Use Committee regulations. Timed pregnant outbred CD1 mice were purchased from Charles River Laboratories, Inc, with the first 24 h of postnatal life designated as P0. We included both male and female mice, aged P30—P60, for this study. For some birth dating experiments as described in the text, pregnant dams were given a single intraperitoneal (i.p.) injection of 5-ethynyl-2′-deoxyuridine (EdU; 30 μg g^−1^ body weight) at E11.5, prior to IUE surgery at E15. Litters were kept in individual ventilated cages until weaning age (P21), after which mice were housed in single-sex groups with 2–5 animals per unit. Cages were maintained in temperature-controlled facilities with 12-h light/12-h dark cycles.

### *In Utero* Intracranial Injection, Electroporation, and Viral Infection

Embryonic cortical injections were performed as part of the IUE procedure during needle insertion into the lateral ventricle (Saito and Nakatsuji 2001; Saito 2006). Embryos were injected once unilaterally between embryonic day 14 and 17 (E14–E17) for all electroporations, as specified in the text. Intracranial injections were not performed at gestational ages below E14 or over E17 due to the technical challenges associated with maintaining embryo viability. To keep consistent experimental design parameters, however, all quantifications for *in vivo* and fixed-tissue analyses used only E15-injected embryos.

All *in utero* injections were targeted toward prospective somatosensory cortices. Injection solutions included the following plasmid and viral components for neuronal labeling: pCAG-tdTomato (based on Addgene plasmid 11 150) and rAAV8-hSyn-eGFP (UNC Vector Core, Lot AV5075D; titer 3.9e12 GC mL^−1^). Injection solutions contained either pCAG-tdTomato (1.5 μg μL^−1^ final concentration) only, or both pCAG-tdTomato (1.5 μg μL^−1^ final concentration) and AAV8-hSyn-eGFP (final titer 3.9e11 GC mL^−1^) in saline solution. All injection solutions contained Fast Green FCF dye (0.2 mg mL^−1^; TCI) to facilitate their visual tracking during the injection procedure. Procedures for *in utero* electroporation have been previously described (Saito and Nakatsuji 2001; Saito 2006). Briefly, timed pregnant CD1 dams were anesthetized with a saline solution containing both ketamine (100–120 mg kg^−1^) and xylazine (10–12 mg kg^−1^), delivered via intraperitoneal injection. Following induction of deep surgical anesthesia, midline incisions (1¼ inch) were made into the abdominal skin and muscle wall to access the underlying uterus. Pulled glass capillary needles (10 μL Drummond Scientific Glass Capillaries, Cat# 3-000-210-G; pulled to ~50 μm diameter at tip and ~125 μm diameter at 1 mm above tip) were then used to puncture the uterine wall and deliver (Picospritzer II, General Valve) 0.5 μL of injection solution into the lateral ventricle of individual embryos. Each embryo was injected only once and subsequently electroporated using BTX tweezertrodes aimed at the somatosensory cortex of the injected hemisphere. All electroporations were conducted using four 50-ms, 50-V electrical pulses, delivered at 1-s intervals (BTX Harvard Apparatus 8300 pulse generator).

### Postnatal Intracranial Viral Injection

Intracranial injections were performed on P0 pups from timed pregnant CD1 dams that were naïve to the *in utero* electroporation procedure. Injection solutions included one of the following viruses to mark injected regions: rAAV8-hSyn-eGFP (UNC Vector Core, Lot AV5075D; titer 3.9e12 GC mL^−1^) and rAAV2-CaMKIIa-mCherry (UNC Vector Core, Lot AV4377d, 3.8e12 titer GC mL^−1^). P0 intracranial injections were performed essentially as previously described (Kim et al. 2013). Briefly, neonates were cryoanesthetized (Phifer and Terry 1986) within 24 h following birth. After confirming loss of voluntary movement, neonates were placed in the prone position on a polymer cooling block. A pulled glass capillary needle (10 μL Drummond Scientific Glass Capillaries, Cat# 3-000-210-G; pulled to ~75 μm diameter at tip and ~325 μm diameter at 4 mm above tip), advanced at a 90° angle from the dorsal scalp through the lateral ventricle to a 4 mm depth, was then used to deliver into the brain parenchyma 1 μL of virus solution (diluted 1:10 in phosphate buffered saline [PBS] and 0.2 mg mL^−1^ Fast Green FCF, from stock). Each neonate received only one unilateral intracranial injection targeted over somatosensory cortices. Injected neonates were then rewarmed on a heating pad and placed back with their biological mother. All P0-injected mice were sacrificed for analysis at P30, with quantifications and analyses performed in fixed tissue to circumvent the obstructive meningeal scarring and parenchymal adhesions associated with P0 injections on *in vivo* imaging.

### Postnatal Subarachnoid Viral Injection

Injection solutions were prepared using AAV9-Syn-GCaMP6f-WPRE-SV40 (Penn Vector Core, Lot CS1001; titer 7.648e13 GC mL^−1^) virus diluted 1:100 in PBS and Fast Green FCF (0.2 mg mL^−1^). P21–P30 mice that had previously been electroporated *in utero* were anesthetized by intraperitoneal injections of ketamine (100 mg kg^−1^) and xylazine (10 mg kg^−1^). The scalp was shaved, cleaned, and then incised to expose the underlying bone. A high-speed drill was next employed to introduce a small burr hole (~0.75 mm diameter) over the transfected hemisphere, taking care to avoid cortical regions suspected to have been directly punctured as part of the *in utero* electroporation procedure. The underlying dura was gently detached, and a 12-μL volume of injection solution (prepared as described above) was infused into the subarachnoid space to achieve viral transfection of both layer I heterotopic and layer II/III neurons via topical cortical application. The scalp incision was then closed with sutures. Cranial windows for *in vivo* imaging were prepared over injected cortical hemispheres 3–4 weeks following the subarachnoid AAV infusion.

### Cranial Window Surgery and *In Vivo* Imaging

All *in vivo* imaging was performed using cranial windows (Hill et al. 2018). Briefly, mice were anesthetized using ketamine (100 mg kg^−1^) and xylazine (10 mg kg^−1^), delivered via i.p. injection. The dorsal skull surgical field was shaved and cleaned, and an approximately 4-mm diameter circular region of skull and dura mater was excised from the injected hemisphere. A #0 transparent glass coverslip was then gently implanted on top of uncovered pial surface to serve as the cranial window. Glue and dental cement were applied to secure the window to the surrounding skull bone.

To detect neuronal cell bodies *in vivo* as described in the text, the fluorescent membrane-permeable probe NeuO (NeuroFluor, Stemcell Technologies Cat# 01801, diluted 1:25 in PBS) was applied to exposed cortex for 20 min followed by a 1–2-min PBS rinse, before the placement of the #0 glass coverslip during the cranial window surgery. In some cases, 100 μL Evans blue (TCI; 1 mg mL^−1^) was injected intravenously after the cranial window surgery to label the cortical vasculature.

Except for GCaMP6f calcium imaging experiments, all *in vivo* imaging studies used mice that were anesthetized via i.p. ketamine and xylazine injection. *in vivo* imaging of anesthetized mice was performed at P30 immediately after cranial window surgery. All intravital GCaMP6f calcium imaging was carried out in P50–P60 awake head-fixed mice starting 4 h after arousal from cranial window surgical anesthesia.

Confocal *in vivo* images of previously injected cortical areas with or without the needle tract sites were acquired using a 20× water immersion objective (Leica, 1.0 NA) on a Leica SP5 upright laser scanning microscope. Spectral confocal reflectance (SCoRe) imaging to detect myelinated axon segments was performed as previously described (Schain et al. 2014; Hill et al. 2018) by capturing the simultaneously reflected light signals from 488, 561, and 633 nm multi-wavelength laser excitation outputs. Single-photon laser outputs were tuned to the following excitation wavelengths for fluorescence imaging: 488 nm for GFP and NeuO; 561 nm for tdTomato and mCherry; 633 nm for Evans blue. Sequential imaging was employed to minimize overlap between SCoRe reflection and individual fluorescence emission signals for all *in vivo* confocal imaging experiments.

Needle tracts from injections performed *in utero* were identified by the abrupt changes in orientations of labeled dendritic and axonal processes around breaks in the cortical surface *in vivo*. Since needle tracts identified in this manner always displayed ectopic neural clusters of similar expanse in layer I, the outer boundaries of “heterotopia” *in vivo* were defined as the layer I needle tract borders for all quantifications. Ipsilateral, noninjected cortical areas were defined as layer I regions at least 150 μm away from a discernable injection site border. Heterotopia and ipsilateral control regions were imaged using identical laser output and image acquisition configurations that were determined for each experimental data set. Confocal z-stacks were acquired at 1024 × 1024 pixel resolution starting from the pia through depths of up to 120 μm below the cortical pial surface. In some cases, time-lapse imaging of *in utero*–induced heterotopia was also performed at 512 × 512 pixel resolution.

For some GCaMP6f calcium imaging experiments that involved visualization of deeper cortical regions in layers II/III as indicated in the text, time-lapse fluorescence images were acquired using a 20× water immersion objective (Zeiss, 1.0 NA) on a Prairie Technologies two-photon microscope fitted with a mode-locked, tunable Spectra Physics Mai-Tai laser. In these experiments, the two-photon laser was tuned to 920 nm for excitation of both GCaMP6f and TdTomato fluorophores. All two-photon time-lapse imaging was performed at 512 × 512 pixel resolution.

### *In Vivo* Image Processing and Quantification

Except for GCaMP6f calcium imaging experiments, which were conducted using P50–P60 mice, all intravital imaging studies were performed using P30 mice, with quantifications carried out on those that had previously been injected at E15. All *in vivo* images were processed and quantified using ImageJ/FIJI.

For SCoRe density quantifications, we analyzed single *z*-sections located 10 μm deep to the cortical pial surface from both heterotopic and ipsilateral control regions. SCoRe density values were assessed using a custom-built, automated thresholding and binarization macro in Image/FIJI, with Robust Automatic Threshold parameters set to noise = 25, lambda = 3, min = 31. Randomly selected, equally sized regions of interest (ROIs) were used to determine SCoRe density values within the centers and edges of the heterotopia versus control regions. A “heterotopion center” was defined as the circular region with a radius extending from the needle tract center to 1/3 of the radius of the needle tract. A “heterotopion edge” was defined as the needle tract concentric circular region just outside the “heterotopion center,” extending from the “heterotopion center” outer edge to the outermost border of the needle tract. Average SCoRe densities were determined for the heterotopion center, heterotopion edge, and control area for each mouse (*n* = 8 mice). Statistical analyses were carried out using Wilcoxon matched pairs, signed-rank nonparametric tests.

For NeuO dye-labeled cell body density quantifications, data were analyzed from the first 75 μm of cortex deep to the pial surface of both heterotopic and control regions in layer I. Equal-sized volumes (50 μm^3^) were randomly selected from the superficial cortical *z*-stacks captured from both heterotopic and control regions. The number of NeuO^+^ cell bodies was manually counted in each volume. Average NeuO^+^ cell body densities were then determined for the heterotopic and control region of each mouse (*n* = 6 mice). Statistical analyses were performed using Wilcoxon matched pairs, signed-rank nonparametric tests.

For quantification of neuronal calcium dynamics in heterotopia versus adjacent noninjected cortical areas, we used the two-photon microscope to capture 150 × 150 μm field of view (FOV) time-lapse images from both layer I heterotopia and noninjected ipsilateral layer II/III regions. Time-lapse imaging (512 × 512 pixel resolution; 2 Hz) was performed in awake, head-fixed mice during a 2-h time window that started 4 h after their arousal from cranial window surgical anesthesia. For heterotopic regions, FOVs were randomly selected from within the first 75 μm below the cortical surface in layer I. For layer II/III regions, FOVs were randomly selected between 120 and 175 μm below the cortical surface. Six to eight separate FOVs (exactly half from layer I heterotopia and half from ipsilateral layer II/III regions) were imaged in each mouse, with each FOV recorded for 120 s per trial for three trials. The order in which FOVs were acquired from heterotopia and layer II/III regions was alternated between mice. Time series analyses were performed using ImageJ/FIJI. Prior to quantifications, TurboReg plugin in ImageJ/FIJI was used to align all time series images in the XY plane.

To quantify GCaMP6f fluorescence changes in individual cells, ROIs were manually selected to encapsulate neuronal cell bodies. GCaMP6f^+^ cell bodies that exhibited tdTomato labeling from the IUE procedure were excluded from all analyses. Approximately 10–25 GCaMP6f^+^ cells were analyzed in each FOV. Baseline GCaMP6f fluorescence intensity values (F) for each cell were defined in each trial as the average of the lowest 20% of the recorded values per trial, with fluctuations from this baseline denoted as Δ*F*/*F*. A spike event was defined in each trial as a Δ*F*/*F* > 0.5. Spike event frequencies were averaged across three trials for each cell in both layer I heterotopia and layer II/III regions for *n* = 7 mice. Statistical analyses were performed using Wilcoxon matched pairs, signed-rank nonparametric tests.

For quantifications of the global synchronization index reflecting the relative coordination of GCaMP6f activity, analyses were performed using Fluorescence Single Neuron and Network Analysis Package (FluoroSNNAP) (Patel et al. 2015). Briefly, this semiautomated software implements a correlation matrix-based algorithm (Li et al. 2007, 2010; Patel et al. 2015) to compute the normalized global synchronization indices (ranging in value from 0 to 1) for time series calcium imaging data of neural populations. The highest value, 1, signifies entirely synchronized firing throughout an identified cluster of neurons, whereas 0 indicates the total absence of synchrony. Synchronization cluster analyses were performed using template-based calcium event detection parameters set to detection threshold = 0.85, minimum size of synchronization clusters = 2, and surrogate resampling = 20. A cluster of neurons was defined as the GCaMP6f^+^ cell population within one FOV. Global synchronization indices were averaged across three trials for each FOV in both layer I heterotopia and layer II/III regions for *n* = 7 mice. Statistical analyses were performed using Wilcoxon matched pairs, signed-rank nonparametric tests.

### Tissue Processing and Immunohistochemistry

At P30, mice were deeply anesthetized using ketamine and xylazine and then perfused transcardially with 4% paraformaldehyde in PBS. Harvested brains were postfixed overnight using the same solution at 4 °C and then vibratome sectioned (coronal; 75 μm thickness) for fixed-tissue analysis.

To identify brain regions that had been injected as part of the IUE procedure, a fluorescence microscope was next used to examine the sections for visibly disrupted layer I regions showing transfected neurons and/or neuronal processes, similar to what has been previously described (Rosen et al. 2007; Wang et al. 2020). The disrupted regions often revealed cellular clusters containing eGFP^+^ neurons that protruded past the pial surface, which were never observed in intact cortical regions or in noninjected mice. Sections containing visibly disrupted layer I cortical cytoarchitecture indicative of injection-associated trauma were then selected for further processing. Immunohistochemistry, performed as described below, was used to confirm the presence of layer I heterotopia at all identified IUE injection sites. Coronal sections from P0-injected mice were screened in a similar manner for injected areas, which were identified by virally transfected axonal fibers and neuronal cell bodies concentrated around visible needle tracts (data not shown).

Prior to immunostaining, all free-floating sections were heated to 95 °C for 30 min in 50-mM sodium citrate buffer (0.05% Tween-20, pH 6.0) for antigen retrieval and elimination of endogenous eGFP expression. After rinsing the sections in PBS at room temperature, EdU labeling was next carried out in some experiments as specified by the Click-iT EdU Alexa Fluor-647 Imaging Kit protocol (Cat# C10340). Before proceeding to antibody staining, all tissues were preincubated for 1–2 hr in 0.1% Triton X-100 and 5% Normal Goat Serum (NGS; Jackson Immunoresearch, Cat# 005-000-121) in PBS at room temperature. Slices were then incubated with primary antibody for 1.5 h–2 days as needed in 0.1% Triton X-100 and 5% NGS in PBS at 4 °C. The primary antibodies used were rabbit anti-NeuN (Abcam, Cat# ab177487, 1:3000), mouse anti-NeuN (Abcam, Cat# ab104224, 1:1000), mouse antimyelin CNPase (clone SMI 91, Biolegend, Cat# 836404, 1:1000), rabbit anti-Cux1 (Novus Biologicals, Cat# NBP2-13883, 1:100), mouse anti-Tle4 (E-10) Alexa Fluor 647 (Santa Cruz Biotechnology, Cat# sc-365406 AF647, 1:100), mouse anti-GAD-67 (EMD Millipore, Cat# MAB5406, 1:1000), rabbit anti-Iba1 (Wako, Cat# 019-19741, 1:600), mouse anti-Aldh1l1 (clone N103/39, NeuroMab, Cat# 75-140, 1:500), chicken antineurofilament NF-H (EnCor, Cat# CPCA-NF-H, 1:500), chicken anti-GFP (Abcam, Cat# 13970, 1:500), and rabbit antimyelin basic protein (MBP; Abcam, Cat# 40390, 1:1000). Following primary antibody incubation, slices were washed in PBS and then incubated with Alexa Fluor dye-conjugated secondary antibodies of the appropriate host species at 1:600 dilution in 0.1% Triton X-100 and 5% NGS in PBS for 1–2 days at 4 °C. After secondary antibody incubation, sections were washed again in PBS and incubated with 2.5 μg mL^−1^ 4′,6-diamidino-2-phenylindole (DAPI) in PBS for 15 min at room temperature to counterstain cell nuclei. Following an additional subsequent wash, stained sections were mounted with 25% mounting media solution (Dako Ultramount, Cat# S1964; diluted 1:4 in PBS) onto glass slides for imaging.

### Fixed-Tissue Imaging and Quantification

Images of stained sections were collected using a Leica SP5 upright confocal laser scanning microscope. Lower magnification views of analyzed sections for presentation were acquired using ×10 and ×20 Leica objectives. All fixed-tissue images for quantification were captured through a ×40 Leica water immersion objective. For immunohistochemical analyses of *in utero*–injected mice, “heterotopia” were defined as the layer I cortical regions demarcated by ectopic NeuN^+^ cell body clusters identified at IUE needle tract sites. For P0-injected mice, because the heterotopia associated with needle tracts were more variable in size, ranging from several cells to 350 μm in diameter (data not shown), data for these injected areas were obtained from 300 μm × 90 μm FOVs centered on visible needle tracts that were within the first 100 μm below the pial surface. Control, noninjected regions were specified on corresponding contralateral cortices at similar mediolateral distances from the midline. Identical laser output and image acquisition configurations were used to capture images of both heterotopia and contralateral control regions throughout each immunostaining set.

All data for Aldh1l1, Iba1, CNPase, and GAD-67 cell density quantifications were acquired from the most superficial 100 μm of cortical tissue of both heterotopic and contralateral homologous control regions in layer I. The number of cell bodies confirmed by DAPI labeling that were positive for each marker was manually counted in randomly selected volumes within z-stacks acquired from both regions. The volumes were kept at identical dimensions between heterotopia and contralateral control regions for each experiment. For GAD-67 immunostaining, the presence of neuronal cell bodies was also confirmed by NeuN labeling. Two coronal sections containing heterotopia were analyzed per animal, with each immunostaining set comprising *n* = 6 animals. Statistical analyses were performed using Wilcoxon matched pairs, signed-rank nonparametric tests.

Data for CNPase density quantifications were acquired from the most superficial 100 μm of cortical tissue in both heterotopia and contralateral control regions. CNPase intensity was assessed using an automated thresholding and binarization plugin in Image/FIJI (Robust Automatic Threshold parameters set to noise = 1, lambda = 2, min = 208) for equally sized ROIs that were randomly selected from within heterotopic and contralateral control *z*-projections (15 μm thickness). CNPase density values for each section were determined by subtracting the automated measurements made in additional background regions from those made in assessed heterotopic and control regions. Two coronal sections containing heterotopia were analyzed per animal (*n* = 6 animals). Statistical analyses were performed using Wilcoxon matched pairs, signed-rank nonparametric tests.

To examine the neuronal composition of the embryonically induced heterotopia, coronal sections were stained for NeuN, DAPI, and one of the other following stains: Cux1, Tle4, or EdU as indicated in the text. Data were acquired from within the most superficial 100 μm of the neocortex for layer I heterotopia and across the entire cortical wall for contralateral homologous control regions. For quantification of layer I heterotopia, equally sized volumes of interest (VOIs) were randomly selected from within the first 100 μm below the pial surface. For quantification of control regions on the contralateral hemisphere, the cortical wall was partitioned into 11 equal volume bins extending from the pial surface to white matter, with Bin1 encompassing layer 1 and Bin 11 adjoining white matter at its lower boundary. For each heterotopic VOI and control bin, the 1) number of NeuN^+^ cells and 2) proportion of NeuN^+^ cells that were also Cux1, Tle4, or EdU positive were manually quantified in ImageJ. Cells were identified as EdU positive if >50% of their nuclear volume, defined by DAPI, was occupied by EdU. Two sections were analyzed per mouse, with sample sizes for each group denoted in the text and figure legends.

The sites of injection analyzed in this study were positioned in somatosensory cortices and immediately adjacent areas in both male and female mice. Given the minimal variation in the morphological appearances of needle tracts between male and female mice and across different cortical areas, we pooled together these variables in our analyses. No animal subjects or experimental data points were excluded from analysis, and the nature of our study did not require subject randomization or experimenter blinding. No statistical methods were used to predetermine sample sizes, although our sample sizes are comparable to those published and generally accepted in the field. GraphPad Prism 7 was utilized for all statistical analyses, and Wilcoxon matched pairs signed-rank nonparametric tests were used to determine statistical significance (declared for *P* values below 0.05; two-tailed) because a normal distribution of differences between the paired data could not be assumed. Data are reported as mean ± SEM, unless otherwise noted.

## Results

### Spatiotemporally Precise Layer I Heterotopia Generation and Visualization in the Live Mouse Brain

**Figure 1:**
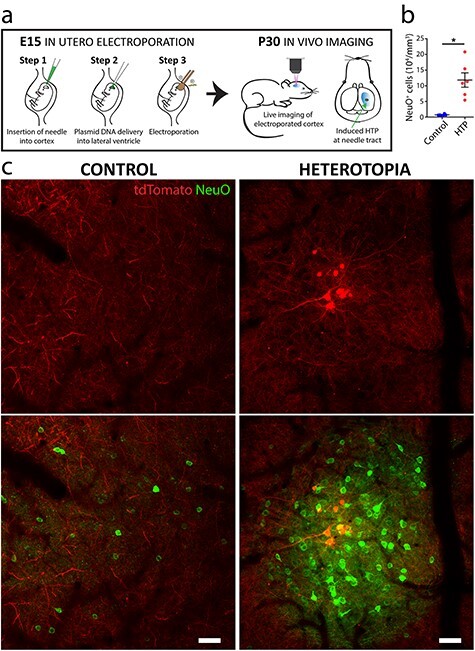
Targeted induction and high-resolution imaging of layer I heterotopia in the live mouse. (*a*) Diagram showing a single neocortical heterotopion induced at the needle tract during *in utero* electroporation (IUE) at embryonic day (E15). The site is relocated postnatally for detailed investigation of the resulting layer I heterotopion by intravital imaging. (*b*) Quantification showing significantly increased neuron density (*n* = 6 mice) within heterotopia compared with ipsilateral neighboring layer I control regions (Wilcoxon nonparametric, matched-pairs signed rank test ^*^P < 0.05). Each point represents the heterotopion or control region from one animal. Horizontal lines and error bars denote mean and SEM, respectively. Descriptive statistics are indicated in Supplementary Table 1. (*c*) *in vivo* fluorescence images of a representative layer I heterotopion (right) and neighboring ipsilateral, noninjected control region in a P30 mouse (left). Notice the tightly packed cluster of fluorescently labeled neuronal cell bodies (NeuO; green). Neurons within the heterotopion can also be readily labeled during the electroporation procedure and visualized *in vivo* (tdTomato; red). In contrast, neurons in more sparsely populated control layer I regions never demonstrate IUE-mediated labeling (left column). HTP, heterotopia. Scale bars, 50 μm (*c*).

*in utero* electroporation (IUE) is a powerful technique that permits labeling and genetic manipulation of targeted cortical neurons (Saito and Nakatsuji 2001; Tabata and Nakajima 2001; Lo Turco et al. 2009). As part of the IUE procedure, a thin glass microcapillary needle is used to deliver genetic material into the lateral ventricle of embryonic stage animals for the electroporation of neuronal progenitors (Fig. 1*a*). While implementing IUE to label and image cortical axons *in vivo*, we unexpectedly found that cortical sites that had been injected during the procedure developed marked accumulations of labeled neuronal cell bodies in layer I of adult mice (Fig. 1*c*, top row, and Supplementary Fig. 1*a*). Electroporated pyramidal cells are normally found in layers II/III of cortical regions; thus, their distinct presence at injected layer I sites stood out in contrast to noninjected surrounding cortical areas (Fig. 1*c*, top row, and Supplementary Fig. 1*a*). *in vivo* labeling of neurons with a fluorescent dye called NeuO (Er et al. 2015) further revealed that the IUE-labeled neuronal cell bodies constituted only a small fraction of the total neuronal cell bodies ectopically positioned (Fig. 1*b* and *c*; for detailed statistics, see Supplementary Table 1; mean diameter of NeuO^+^ ectopic cell clusters 474 ± 174 μm SD in *n* = 23 mice). Abnormal superficial clustering of neuronal cell bodies is a defining feature of layer I heterotopia (Jacob 1940; Morel and Wildi 1952; Schulze and Braak 1978; Kaufmann and Galaburda 1989; Meencke and Veith 1992; Yamamoto et al. 1997), a subtype of neocortical heterotopia that has been linked to learning impairments in humans (Galaburda and Kemper 1979; Galaburda et al. 1985; Humphreys et al. 1990). Their presence demonstrates that direct injections into the cortex performed as part of the IUE procedure can induce neocortical heterotopia amenable to precise molecular and cellular studies in vivo*.*

### Aberrant Axonal and Myelin Patterns Characterize Layer I Heterotopia Formed during a Critical Embryonic Time Window

**Figure 2:**
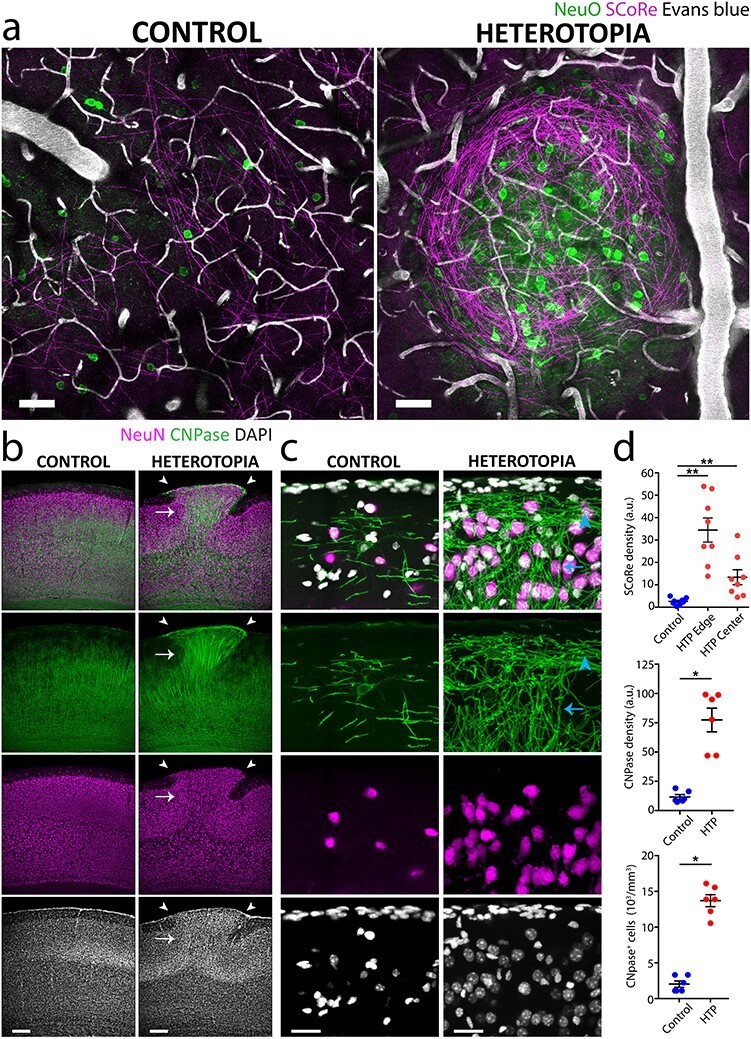
Heterotopia contain an abundance of myelinated axons. (*a*) *In vivo* combined fluorescence and label-free SCoRe myelin images of a representative induced heterotopion (right; same as in Fig. 1*c*) and neighboring ipsilateral, noninjected control region (left) in a P30 mouse. The tightly packed heterotopic neuronal cell bodies (NeuO) are surrounded by a circumscribed abundance of aberrantly projecting, myelinated axon segments (SCoRe). (*b*, *c*) Low magnification (*b*) and high magnification (*c*) immunostainings of an induced heterotopion and its corresponding contralateral control region taken from a P30 mouse, showing increased oligodendrocyte CNPase expression associated with the heterotopion, as defined by the ectopically positioned NeuN^+^ neuronal cell bodies in layer I (*b*, white arrowheads). Notice the more horizontal orientation of myelin segments located closer to the pial surface of the heterotopion (*c*, blue arrowhead). Deeper myelin segments, in contrast, are organized in a more radial fashion (*c*, blue arrow) and fasciculate into densely myelinated fiber bundles that project through lower cortical layers (*b*, white arrow). (*d*) Quantifications of SCoRe *in vivo* (*n* = 8 mice; top) and CNPase in fixed tissue (*n* = 6 mice; middle and bottom), showing increased myelination and oligodendrocyte cell body densities in induced layer I heterotopia compared with noninjected contralateral control regions. Each point represents the layer I heterotopion or control region from one animal. Horizontal lines and error bars denote mean and SEM, respectively (Wilcoxon matched-pairs signed rank nonparametric test; ^*^P < 0.05, ^*^^*^P < 0.01). HTP, Heterotopia. Descriptive statistics are given in Supplementary Table 1. Scale bars, 50 μm (*a*), 200 μm in (*b*), and 50 μm (*c*).

**Figure 3:**
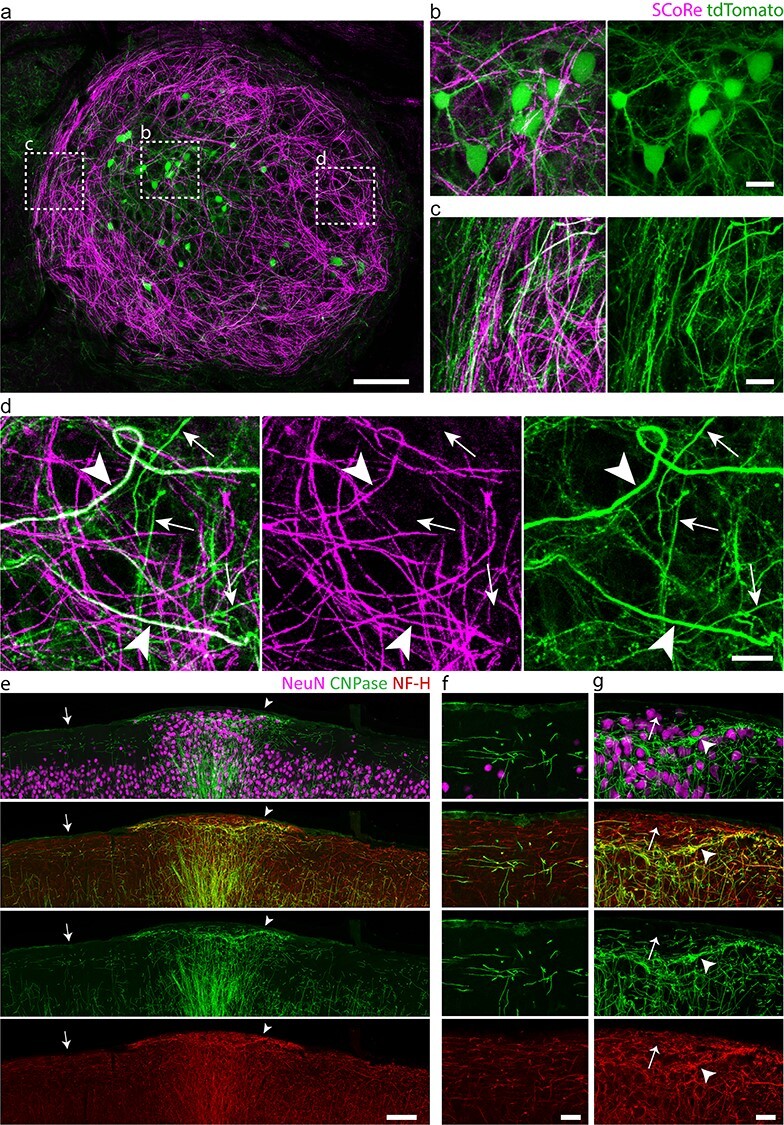
Myelinated and unmyelinated axons follow aberrantly looping and concentric paths. (*a*–*d*) Intravital imaging of a nest-like heterotopion at P30 in (*a*) using SCoRe and confocal fluorescence microscopy showing *b*, labeled neuronal cell bodies (tdTomato; green) dispersed throughout the aberrantly oriented myelinated fibers (SCoRe; magenta) and *c*, winding axonal projections that occasionally fasciculate into bundles at the edges of the heterotopion. (*d*) Myelinated (arrowheads) and unmyelinated axon segments (arrows) are observed within the heterotopion. (*e*–*g*) Low-magnification (*e*) and high-magnification (*f*, *g*) immunostainings of an embryonically induced heterotopion (*g*), confirming the presence of both myelinated (*g*, arrowhead) and unmyelinated (*g*, arrow) axon segments (*f* and *g* show high-magnification images of areas indicated by arrow and arrowhead in *e*, respectively). NF-H, neurofilament heavy chain. Images are representative of experiments performed in at least six animals. Scale bars, 100 μm (*a*), 15 μm (*b*–*d*), 100 μm (*e*), and 25 μm (*f*, *g*).

Using this methodology, we set out to examine the cellular composition of the layer I heterotopia. Interestingly, we observed markedly aberrant patterns of myelinated axons exclusively associated with the ectopic neuronal clusters (Figs 2*a* and 3*a*, Supplementary Fig. 1*a,b*, and Supplementary Videos 1 and 2), as visualized *in vivo* by SCoRe microscopy, a technique that enables high-resolution label-free imaging of myelinated axons (Schain et al. 2014; Hill et al. 2018). This was further corroborated by fixed-tissue immunohistochemistry of myelin markers, showing increased oligodendrocyte and myelin densities at the sites of injection (Figs 2*b–d* and 3*e–g*, and Supplementary Fig. 1; for detailed statistics, see Supplementary Table 1). On closer examination *in vivo*, we observed both myelinated and unmyelinated axons swirling in a nest-like fashion around heterotopic neurons (Figs 2*a* and 3*a–d*), often forming thick concentric borders that were most prominent toward the pial surface (Fig. 3*e–g*, and Supplementary Fig. 1 and Supplementary Video 1). Radially oriented, myelinated fiber bundles were also observed projecting out from underneath the heterotopia (Fig. 2*b*,*c* and Supplementary Fig. 2), similar to previous descriptions of spontaneously occurring layer I heterotopia (Jacob 1940; Morel and Wildi 1952; Sherman et al. 1990; Ramos et al. 2008; Lipoff et al. 2011). Thus, by applying our methodology in combination with SCoRe microscopy and immunohistochemical analyses, we discovered that distinct axon guidance and myelin abnormalities occur in embryonically induced layer I heterotopia.

**Figure 4:**
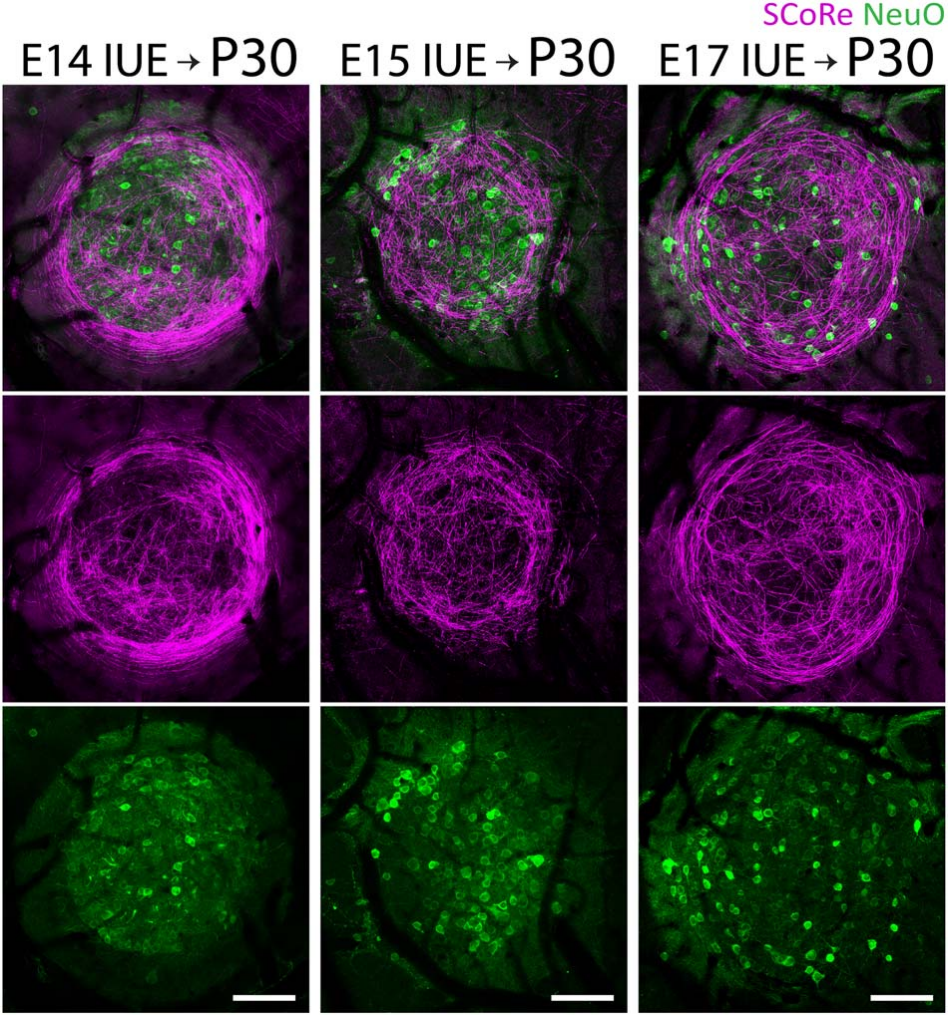
Heterotopia can be induced at various embryonic stages of corticogenesis. *In vivo* NeuO dye neuron staining (green) and SCoRe myelin imaging (magenta) of P30 mouse cortices that were previously electroporated at E14 (left column), E15 (middle column), and E17 (right column), all showing similar aberrantly projecting, myelinated axon segments encircling the ectopic neuron clusters. Images are representative of observations made in at least three animals per IUE-injected age group. Scale bars, 100 μm.

**Figure 5:**
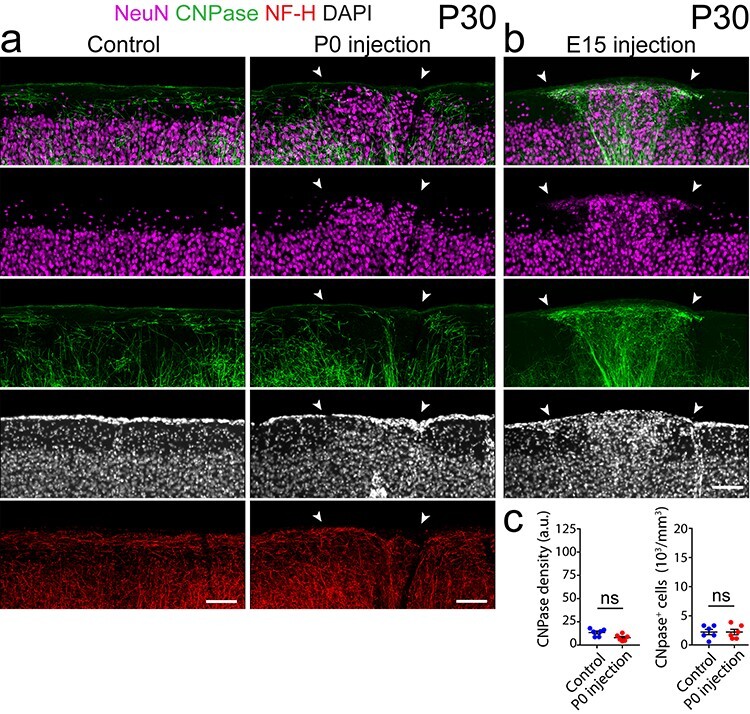
Postnatally induced heterotopia do not develop aberrant axons or myelination. (*a*) Confocal images of an immunolabeled coronal section from P30 mouse cortex, showing no changes in myelination (CNPase) or axonal pathfinding (NF-H) associated with a P0-induced heterotopion (NeuN; arrowheads), compared with its contralateral control region (left). (*b*) In contrast, neuronal heterotopia induced *in utero* at E15 (NeuN; arrowheads) show dramatic changes in local layer I myelin (CNPase) expression and patterning. (*c*) Quantifications of CNPase expression, showing no significant differences in the myelin or oligodendrocyte cell body densities (*n* = 6 animals) between postnatally induced heterotopia and control regions. Each point represents the P0-induced layer I heterotopion or control region from one animal, with horizontal lines and error bars denoting mean and SEM, respectively. Wilcoxon matched-pairs signed rank nonparametric test was used to determine significance; NS, no significance. NF-H, neurofilament heavy chain. Descriptive statistics are given in Supplementary Table 1. Scale bars, 100 μm (*a*, *b*).

To investigate whether the marked axonal and myelin changes were dependent on induction of heterotopia at specific developmental ages, we performed IUE at various time points during cortical development between embryonic days 14 (E14) to E17 and postnatal injections at P0. We found that whereas embryonic injections always resulted in similar nest-like patterns of densely myelinated fiber bundles around heterotopic neurons (100% frequency in *n* > 30 mice; Figs 4 and 5*b*), P0 injections did not result in robust axon guidance or myelination abnormalities, despite the presence of ectopic neural clusters (Fig. 5*a*). Although postnatally induced heterotopia tended to be smaller and more variable in size (data not shown), the axonal and myelin abnormalities were never observed even around larger P0-induced clusters (Fig. 5*a*). Consistent with this, we found no significant difference in the myelin or oligodendrocyte cell body densities (Fig. 5*c*; for detailed statistics, see Supplementary Table 1) between heterotopia and contralateral control regions of P0-injected mice. Likewise, immunolabeling for neurofilament heavy chain (NF-H) did not reveal aberrant accumulations of axonal fibers (Fig. 5*a*). Together, our data indicate previously unrecognized differences in the cellular composition and organization of neocortical heterotopia based on the timing of their induction.

### Astrocyte and Microglia Cell Densities Are Not Altered in Induced Heterotopia

**Figure 6:**
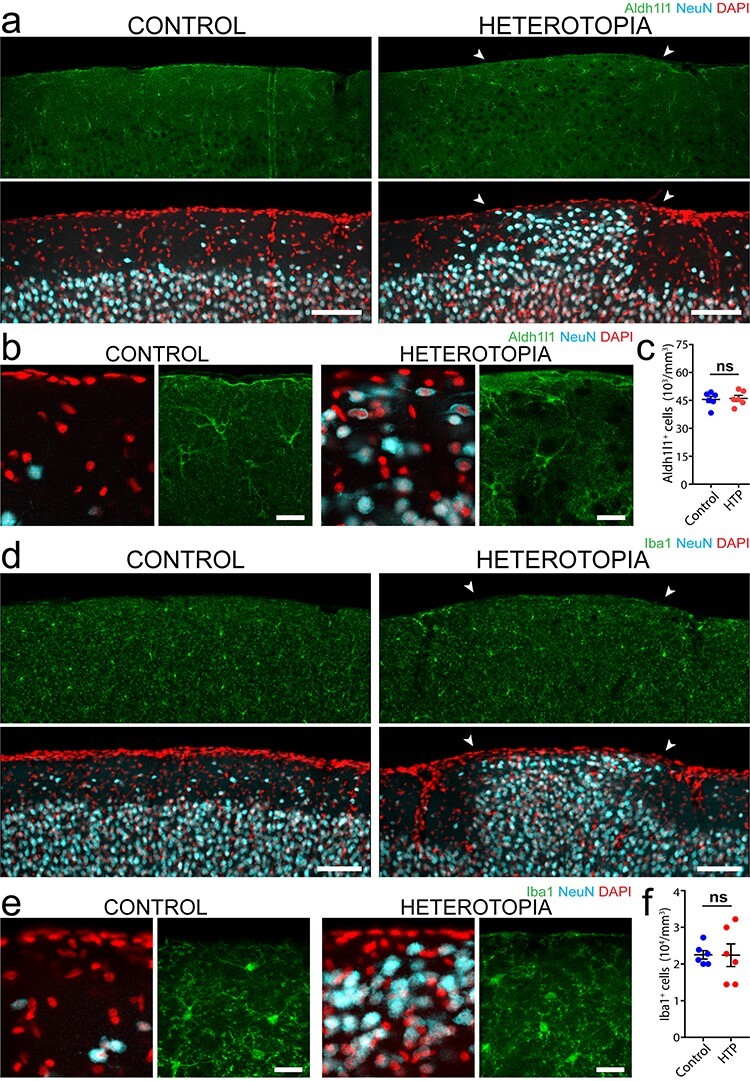
Astrocytes and microglia retain a normal density despite aberrant axonal and myelin distribution. (*a, b*) Low-magnification (*a*) and high-magnification (*b*) images of Aldh1l1-positive astrocytes in P30 mouse cortex, showing similar cell densities in layer I heterotopia (right images, arrowheads) compared with corresponding contralateral control regions (left images). (*c*) Quantifications using P30 mouse forebrain tissue showing no significant difference in astrocyte cell density (Wilcoxon nonparametric matched-pairs, signed rank test, *n* = 6 animals; ns, no significance). (*d, e*) Low-magnification (*d*) and high-magnification (*e*) images of Iba1-immunolabeled P30 mouse forebrain, showing no difference in microglia cell densities between heterotopia (right images, arrowheads) and contralateral control regions (left images). (*f*) Quantifications using P30 mouse forebrain tissue demonstrating no significant difference in microglia cell density between layer I heterotopia and corresponding contralateral control regions (Wilcoxon matched-pairs, signed rank nonparametric test, *n* = 6 animals; ns, no significance). For graphs in (*c*) and (*f*), each point represents an embryonically induced layer I heterotopion or control region from one animal, with horizontal lines and accompanying error bars denoting mean and SEM, respectively. Descriptive statistics are given in Supplementary Table 1. HTP, heterotopia. Scale bars, 100 μm (*a*), 20 μm (*b*), 100 μm (*d*), and 20 μm (*e*).

Given the striking abnormalities in oligodendrocyte density, myelination and axon pathfinding observed in the embryonically induced heterotopia, we next wondered whether other glial cell types such as astrocytes and microglia also exhibited altered morphology or density. Immunolabeled cortical brain sections against Aldh1l1 revealed no significant difference in the astrocyte cell density between layer I heterotopia and corresponding contralateral control regions (Fig. 6*a–c*; for detailed statistics, see Supplementary Table 1). Similarly, using Iba1 immunolabeling, we found no regional differences in microglia density or morphology (Fig. 6*d–f*; for detailed statistics, see Supplementary Table 1). Moreover, there was no evidence of pronounced astrocytic or microglial accumulation or altered cellular morphology at the borders of the heterotopia suggesting that a glial scar had not formed due to the embryonic injection. These data suggest that while heterotopia induce marked oligodendrocyte generation, astrocytes and microglia are not significantly influenced by potential local factors derived from ectopic neuronal clusters.

### Heterotopia Contain a Variety of Excitatory and Inhibitory Neurons Born at Different Embryonic Ages

**Figure 7:**
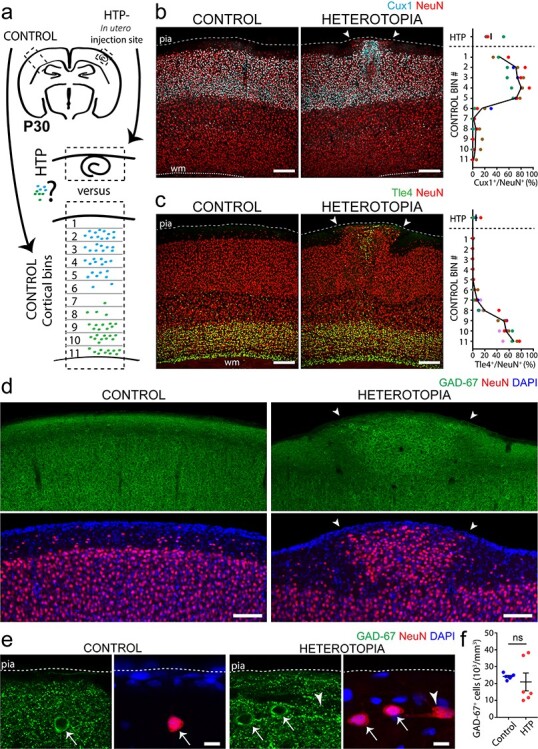
Heterotopia contain a mixed population of excitatory and inhibitory neurons. (*a*) Approach for quantifying layer-specific neuronal marker expression (symbolized by blue and green dots) in layer I heterotopia and across corresponding contralateral control cortices in P30 mice. Contralateral control cortices are subdivided into 11 equally sized bins that together span the entire thickness of the cortex. (*b, c*) Both Cux1^+^ (*b*, cyan) and Tle4^+^ (*c*, green) neuronal cell bodies occur within layer I heterotopia (right images, arrowheads). In contralateral control regions (left images), Cux1 is concentrated in more superficial cortical areas corresponding to layers II/III, whereas deeper areas corresponding to layers V/VI encapsulate the majority of Tle4 labeling. Quantifications (far right) show the percentages of NeuN^+^ cell bodies that also express Cux1 (top) or Tle4 (bottom) within heterotopia and across corresponding contralateral control bins. Each dot corresponds to the layer I heterotopion or control bin of a single animal, with all dots of the same color belonging to the same animal (*n* = 4 animals for Cux1; *n* = 5 animals for Tle4). The black line denotes the mean. WM, white matter. *d*,*e* Low- (*d*) and high-(*e*) magnification z-projections of GAD-67-immunostained P30 mouse forebrain, showing the presence of GAD-67-labeled cells and puncta in both layer I heterotopia (right images, arrowheads) and corresponding contralateral control regions (left images). *f*, Quantifications from P30 mouse tissue showing no significant difference in GAD-67^+^ neuronal cell density between layer I heterotopia and contralateral control regions (Wilcoxon matched-pairs, signed rank nonparametric test, *n* = 6 animals; ns, no significance). Each point represents the layer I heterotopion or contralateral control region from one animal, with horizontal lines and accompanying error bars denoting mean and SEM, respectively. HTP, heterotopia. Descriptive statistics are given in Supplementary Table 1. Scale bars 200 μm (*b*, *c*), 100 μm (*d*), and 10 μm (*e*).

To characterize the neural composition of the embryonically induced heterotopia, we examined the expression of neuronal subtype markers Cux1, Tle4, and GAD-67 (Fig. 7). Cux1 is a transcription factor predominantly expressed in callosal projection neurons in layers II–IV (Molyneaux et al. 2009), whereas Tle4 is primarily restricted to deeper corticothalamic projection neurons of layers V and VI (Molyneaux et al. 2015; Sorensen et al. 2015). We found both Cux1^+^ and Tle4^+^ neurons present to varying degrees in all layer I heterotopia, consistent with a mixed population of excitatory projection neurons from different cortical layers (Fig. 7*b*,*c* and Supplementary Fig. 3). Further analyses revealed the presence of GAD-67^+^ interneurons, which occurred at similar densities within heterotopia as in corresponding layer I control regions (Fig. 7*d–f*; for detailed statistics, see Supplementary Table 1). In addition, layer I heterotopia always contained GAD-67^+^ puncta, consistent with inhibitory synapses (Fig. 7*d*,*e*). Together, our findings suggest that embryonically induced layer I heterotopic neural clusters comprise a diverse cohort of glutamatergic and GABAergic neurons, in line with the heterogeneous population of neuronal subtypes previously described in those occurring spontaneously (Gabel and LoTurco 2001; Ramos et al. 2008, 2014; Gabel 2011).

We next used birthdating techniques to determine whether neurons within the induced heterotopia originate at similar or different time points during development. EdU pulse labeling at E11.5 revealed small but distinct populations of EdU^+^NeuN^+^ cells in all embryonically-generated layer I heterotopia (Supplementary Fig. 4), suggesting that heterotopia contain neurons born during early corticogenesis (Angevine and Sidman 1961; Raedler and Raedler 1978). We also observed cells “birthdate-labeled” via IUE at E15 in heterotopia (~90% heterotopia in *n* > 30 mice; Fig. 1*c*), suggesting the additional presence of later-born neurons (Lo Turco et al. 2009). These data indicate that induced heterotopia contain neurons born at various stages of cortical development.

### Heterotopic Neurons Exhibit Similar Calcium Dynamics as Neighboring Normotopic Neurons

**Figure 8:**
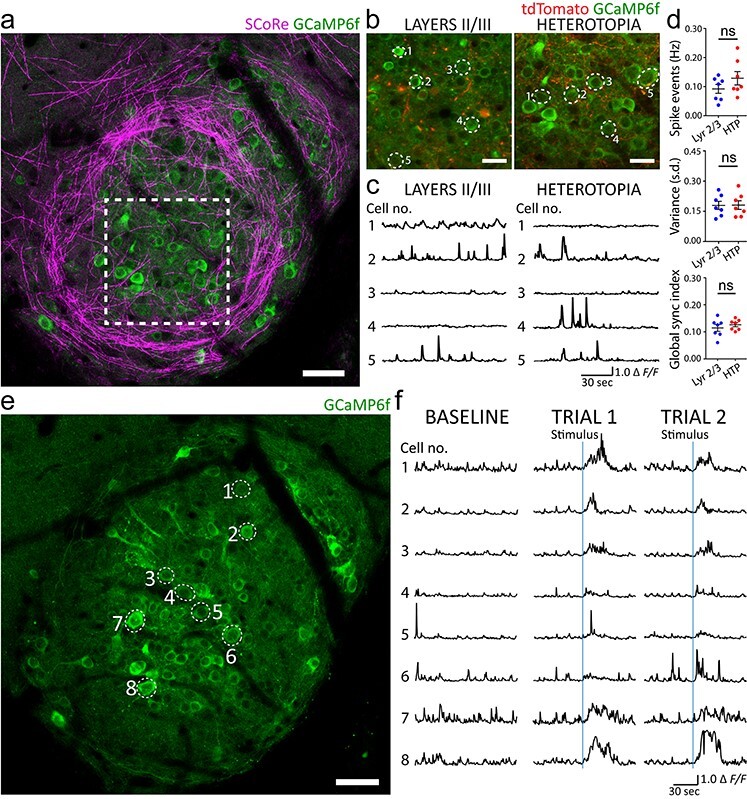
Heterotopic neurons display calcium dynamics similar to adjacent layer II/III neurons and respond to sensorimotor input. (*a*–*c*) *In vivo* time-lapse imaging of layer I heterotopic neurons expressing the calcium sensor GCaMP6f in an awake, head-fixed mouse. Dotted lines in (*a*) indicate an analyzed heterotopic region that is displayed in (*b*). (*c*) Example traces of spontaneous neuronal calcium transients from layer I heterotopic (right) and ipsilateral layer II/III nonheterotopic (left) regions displayed in (*b*). (*d*) Quantifications showing no significant difference in calcium spike event frequency (Hz), variance (SD), or global synchronization index between layer I heterotopia and adjacent layer II/III nonheterotopic regions. Each dot corresponds to the average value for each measure in a layer I heterotopion or nonheterotopic layer II/III region from a single animal. Data represent 493 layer I heterotopic neurons and 594 layer II/III neurons from *n* = 7 mice. Wilcoxon matched-pairs, signed rank nonparametric test was used for all three quantifications; ns, no significance; HTP, heterotopia. Descriptive statistics are given in Supplementary Table 1. (*e*) *In vivoz*-projection of the same GCaMP6f-transfected heterotopion displayed in (*a*), captured at a different depth, showing neurons that were imaged during brief applications of whisker stimulation. (*f*) Baseline calcium dynamics (left column) and calcium fluctuations (middle and right columns) of individual heterotopic neurons in response to stimuli (denoted by solid blue line in two trials) obtained from the awake, head-fixed mouse imaged in (*e*). Experimental observations in (*e*, *f*) were replicated in three mice. All data and images from (*a*–*f*) were obtained from P50 to P60 mice with heterotopia induced at E15 and virally transfected with AAV-GCaMP6f at P21-P30. Some neuronal cell body and axonal labeling by pCAG-TdTomato (red) is the result of E15 IUE-mediated transfection. Scale bars, 50 μm (*a*), 25 μm (*b*), and 50 μm (*e*).

Despite their linkage to a wide spectrum of neurological disorders, the cellular dynamics and mechanisms by which individual heterotopic cells contribute to disrupted neural circuit functioning are poorly understood. We examined the calcium activity of single cortical neurons in embryonically induced heterotopia and in surrounding, noninjected cortical layer II/III regions of awake head-fixed mice using the genetically encoded calcium indicator, GCaMP6f. Surprisingly, although we found highly variable calcium spike patterns of individual cells within heterotopia (Fig. 8*a–c* and Supplementary Video 3), there were no significant differences in their overall calcium spike event frequency, variance, or synchrony compared with surrounding nonheterotopic layer II/III regions (Fig. 8*d*; for detailed statistics, see Supplementary Table 1). Furthermore, we did not identify any epileptiform activity in our imaging sessions, although we cannot rule out that continuous recordings could have revealed sporadic aberrant activity (Fig. 8*a–c* and Supplementary Video 3). Interestingly, we found that mice that had been startled with brief whisker stimulation consistently responded via neuronal calcium spikes within layer I heterotopia (Fig. 8*e*,*f* and Supplementary Video 4). This result indicates that heterotopic neurons are connected to other brain areas and could thus influence network function.

## Discussion

Here, we describe an inducible model that allows the visualization of focal heterotopia at cellular resolution in the live animal. Building upon previous models, this system employs *in utero* cortical microinjections to induce layer I heterotopia that closely recapitulate those seen in humans (Jacob 1940; Schulze and Braak 1978; Galaburda et al. 1985; Kaufmann and Galaburda 1989; Humphreys et al. 1990; Meencke and Veith 1992; e.g., see Fig. 7 in Jacob; Fig. 1*b* in Humphreys et al.; Fig. 3 in Meencke et al.) and in spontaneous animal models (Sherman et al. 1985; Sherman et al. 1987; Ramos et al. 2008; Lipoff et al. 2011; Toia et al. 2017; e.g., see Fig. 1 in Sherman et al. 1987; Fig. 1 in Ramos et al; Fig. 1 in Lipoff et al.). Pairing this methodology with intravital fluorescence and label-free (SCoRe) microscopy of neuronal cell bodies, axons and myelin, as well as time-lapse axonal and calcium imaging (Saito and Nakatsuji 2001; Lo Turco et al. 2009; Schain et al. 2014; Er et al. 2015; Hill et al. 2018), enables detailed studies of heterotopia in the intact brain.

Many animal models of focal heterotopia have been described; however, most involve subcortical or intrahippocampal heterotopia (Riggs et al. 1956; Singh 1977; Amano et al. 1996; Lee et al. 1997; Bai et al. 2003), which are not easily amenable to intravital optical imaging due to their distance from the cortical surface. Moreover, other animal models of more superficially occurring heterotopia (Sherman et al. 1987; Rosen et al. 1992, 2007; Ferrer et al. 1993; Brunstrom et al. 1997) have not been imaged successfully at high resolution *in vivo* mainly as a result of their temporally and spatially unpredictable nature and/or lack of visible cell transfection. Our methodology improves upon these limitations by employing minimally invasive microinjections to both induce layer I heterotopia and deliver plasmid DNA and/or viral vectors for cellular genetic manipulation in the live mouse.

Using our model, we revealed several striking characteristics of neocortical heterotopia. First, we discovered that axonal projections within layer I heterotopia formed swirled and concentric morphologies specifically around heterotopic neuronal clusters. These aberrantly projecting swirling axons were associated with radially oriented fibers extending through the base of the heterotopia, consistent with previous fixed-tissue studies of spontaneously occurring heterotopia (Sherman et al. 1990; Ramos et al. 2008). Interestingly, there was no evidence of glial scarring or mechanical tissue barriers surrounding the heterotopia, suggesting that the directional changes in axon pathfinding are instead likely mediated by disruptions in the precise balance of repulsive and attractive gradients of local axon guidance cues (Métin et al. 1997; Richards et al. 1997; Bagnard et al. 1998; Gao et al. 1998; Polleux et al. 1998). Future studies of the individual neuronal identities of the aberrantly projecting axons, which could derive from various cortical and subcortical sources (Jenner et al. 2000; Dwyer et al. 2011; Gabel 2011; Ramos et al. 2014), may shed light on the possible molecular factors leading to the disrupted axon pathfinding.

The axon guidance abnormalities led us to investigate whether the developmental timing of heterotopia induction could modulate their formation. We found that whereas embryonically induced heterotopia always displayed distinct axon guidance abnormalities, similar defects were not observed in postnatally induced heterotopia. One potential explanation for this surprising finding is the presence of a critical developmental time period during which cortical axon pathfinding is uniquely sensitive to local environmental disturbances. The distinct responsiveness of axons during this critical period could be mediated by the enhanced expression of axon growth cone receptors that increase the ability of axons to extend toward guidance cues (Shewan et al. 2002; Baudet et al. 2012). However, developmentally modulated changes in the signaling gradients of guidance cues themselves could also play a role (Skaliora et al. 1998). An alternative explanation is the induction of subtle glial alterations that could mechanically impair axon outgrowth, although the lack of glial changes in our images does not favor this hypothesis.

In addition to the aberrant axonal organization, we used label-free SCoRe *in vivo* imaging to discover focally increased myelination specifically within the heterotopia. This striking change was due to an increased density of local myelinating oligodendrocytes that deposited myelin sheaths primarily along the aberrantly projecting and concentric axons. Interestingly, the abundant myelin phenotype was only observed in embryonically induced but never in postnatally induced heterotopia. Similar to the critical period for disrupted axonal patterning, these data suggest that the excess myelination seen in embryonically induced heterotopia is instructed by developmentally regulated molecules or biophysical cues, which could accelerate the local production of myelin by stimulating oligodendrocyte precursor cell recruitment and/or differentiation into mature oligodendrocytes. Myelin formation is influenced by axon caliber (Sturrock 1980) and genetic manipulations increasing axon caliber can trigger oligodendrocyte precursor cell proliferation and the ensheathment of classically unmyelinated fibers (Goebbels et al. 2017). Thus, axon caliber could serve as one mechanism that differs between the aberrantly projecting axons found in embryonically induced heterotopia compared with adjacent nonmyelinated fibers. Other potential causes for the increased myelin deposition around heterotopic axons could include cell surface markers or molecular identity. Specific subpopulations of excitatory (Tomassy et al. 2014) and inhibitory (Micheva et al. 2016) neurons exhibit variable myelination patterns, seemingly not directly linked to axon caliber, but instead potentially due to their differential expression of adhesion molecules or altered patterns of neuronal activity. Our methodology now provides a means to test these and other possibilities in precise, real-time live animal studies.

Despite the striking axon and myelin abnormalities associated with the induced heterotopia, we found no evidence of spontaneous epileptiform activity occurring in heterotopic regions or nearby nonheterotopic cortices of awake animals, consistent with previous studies (Roper et al. 1995; Gabel and LoTurco 2001; Ishii et al. 2015). Moreover, spontaneous calcium fluctuations and the relative synchrony of firing between neurons in layer I heterotopia and neighboring layer II/III cortex were similar. This lack of observable difference in activity may reflect the similar neural and glial composition of both brain areas, which could enable a similar balance of excitatory and inhibitory inputs to these regions. Indeed, we identified microglia, astrocytes, Cux1^+^ cells, GABAergic neurons, and GABAergic puncta in layer I heterotopia that are also prevalent in layers II/III (Chmielowska et al. 1988; Nieto et al. 2004; Molyneaux et al. 2009; Meyer et al. 2011; Farhy-Tselnicker and Allen 2018; Jara et al. 2019). A second, nonmutually exclusive possibility is that more metabolically demanding conditions are required to trigger aberrant activity in mice with induced heterotopia. Consistent with this hypothesis, the application of normally subthreshold doses of convulsant drugs can provoke epileptiform activity in mice with heterotopia in vitro (Gabel and LoTurco 2002) and *in vivo* (Gabel and LoTurco 2002; Manent et al. 2009; Ishii et al. 2015). Other animal models of heterotopia, such as the *tish* mutant (Lee et al. 1997) and Ihara’s genetically epileptic rat (Amano et al. 1996) have documented spontaneous epileptiform activity *in vivo*. Reasons for this difference from our findings remain unclear; however, they may be related to disparities in the number, location, or size of heterotopia, or unknown off-target effects of the inherited mutations themselves in the genetic models.

There is little known about the functional dynamics of individual heterotopic neurons during behavioral stimulation in the live animal. Using time-lapse calcium imaging, we revealed that heterotopic neurons display robust responses following brief whisker stimulation in awake mice, consistent with the functional connectivity implicated by previous anatomical, electrophysiological, and behavioral studies (Lee et al. 1997; Chevassus-Au-Louis, Congar, et al. 1998a; Chevassus-Au-Louis, Rafiki, et al. 1998b; Jenner et al. 2000; Gabel and LoTurco 2001; Schottler et al. 2001; Ishii et al. 2015). This functional connectivity suggests that, even though we did not detect aberrant spontaneous activity within heterotopia, these neurons are integrated into the local neural network and are potentially capable of altering neural network function at baseline or under metabolically demanding conditions. There remains considerable debate about the exact role for layer I heterotopia in neurological disorders (Galaburda and Kemper 1979; Galaburda et al. 1985; Kaufmann and Galaburda 1989; Humphreys et al. 1990; Meencke and Veith 1992; Barkovich 1998). They may provoke more subtle cognitive and behavioral disturbances, as implicated by their widely distributed axon projections throughout both ipsilateral and contralateral cortical layers (Jenner et al. 2000; Gabel 2011; Ramos et al. 2014), and subcortical structures (Jenner et al. 2000; Ramos et al. 2014). Indeed, their presence has been linked to reading disability in humans (Galaburda and Kemper 1979; Galaburda et al. 1985; Humphreys et al. 1990) and changes in perceptual and mnemonic abilities in rodents (Waters et al. 1997; Clark et al. 2000; Frenkel et al. 2000; Hyde et al. 2000, 2001; Peiffer et al. 2001). There is evidence that the functional outcomes associated with individual heterotopia may vary with respect to their intracortical positioning (Hyde et al. 2001), which could be related to underlying differences in neuronal connectivity (Jenner et al. 2000; Ramos et al. 2014) or composition (Ramos et al. 2008).

Previous genetic and neuropathological studies (De Bernabé et al. 2002; Halfter et al. 2002; Moore et al. 2002; Devisme et al. 2012; Myshrall et al. 2012; Radner et al. 2013) have suggested that layer I heterotopia arise from acquired defects in the integrity of pial basement membranes during corticogenesis (Nakano et al. 1996; Yamamoto et al. 1997; Romero et al. 2018). Consistently, we found that layer I heterotopia form only at pial surface sites that are previously punctured *in utero*, and never in noninjected cortical regions of CD1 mice, in accordance with the findings of previous studies (Rosen et al. 1992; Ramos et al. 2008). A breach in the pial basement membrane during corticogenesis could trigger the inappropriate migration of diverse neuronal subtypes into layer I by causing the displacement of local anchoring radial glial foot processes, which normally serve as scaffolds for radially migrating neuroblasts during cortical development (Rakic 1972; Nadarajah and Parnavelas 2002). This hypothesis is supported by histopathological studies that have revealed the protrusion of radial glial fibers through breaks in the pial basement membrane that coincide with the location of layer I heterotopia in both spontaneous and mutant animal models (Sherman et al. 1992; Myshrall et al. 2012). Spontaneously occurring heterotopia in rodents (Sherman et al. 1990; Ramos et al. 2008; Lipoff et al. 2011) and in humans (Jacob 1940; Morel and Wildi 1952) display aberrant accumulations of superficial myelinated axons and radially oriented axon bundles that resemble those resulting from embryonic cortical injections (e.g., see Fig. 3 in Jacob; Fig. 8 in Morel et al.; Fig. 8 in Ramos et al. in comparison to induced heterotopia shown here, Fig. 2 and Supplementary Fig. 2). Future studies using our model, including at earlier developmental time points, may provide additional information about the cellular and molecular mechanisms by which layer I heterotopia form and lead to aberrant axon and myelin patterns, as well as their functional implications.

## Author Contributions

A.M.L. and J.G. conceived the initial project. A.M.L., R.A.H, and J.G. designed the experiments. A.M.L. performed all experiments, analyzed the data, and prepared the figures. A.M.L., R.A.H., and J.G. wrote the manuscript.

## Funding

U.S. National Institutes of Health (R01NS089734, R21NS088411, R21NS087511 to J.G.; R00NS099469, P20GM113132 to R.A.H.; National Institute of Neurological Disorders and Stroke Training Grant T32NS007224, and Medical Scientist Training Program Training Grant T32GM007205 to A.M.L.); Donors Cure Foundation (New Vision Award to R.A.H.); National Multiple Sclerosis Society (#RR-1602-07686 to J.G.).

## Notes

We thank F. Chen for his guidance with *in utero* electroporation techniques. *Conflicts of Interest*: The authors declare no conflict of interest.

## Supplementary Material

Supplementary_Data_final_bhab090

Video_1_bhab090

Video_2_bhab090

Video_3_bhab090

Video_4_bhab090

Supp_Video_Legends_bhab090

## References

[ref1] Amano S , Ihara N, Uemura S, Yokoyama M, Ikeda M, Serikawa T, Sasahara M, Kataoka H, Hayase Y, Hazama F. 1996. Development of a novel rat mutant with spontaneous limbic-like seizures. Am J Pathol. 149:329–336. 8686757 PMC1865209

[ref2] Angevine JB , Sidman RL. 1961. Autoradiographic study of cell migration during histogenesis of cerebral cortex in the mouse. Nature. 192:766–768. 10.1038/192766b0 17533671

[ref3] Bagnard D , Lohrum M, Uziel D, Püschel AW, Bolz J. 1998. Semaphorins act as attractive and repulsive guidance signals during the development of cortical projections. Development. 125:5043–5053. 9811588 10.1242/dev.125.24.5043

[ref4] Bai J , Ramos RL, Ackman JB, Thomas AM, Lee RV, LoTurco JJ. 2003. RNAi reveals doublecortin is required for radial migration in rat neocortex. Nat Neurosci. 6:1277–1283. 14625554 10.1038/nn1153

[ref5] Barkovich A , Gressens P, Evrard P. 1992. Formation, maturation, and disorders of brain neocortex. Am J Neuroradiol. 13:423–446. 1566709 PMC8333199

[ref6] Barkovich AJ . 1998. Neuroimaging manifestations and classification of congenital muscular dystrophies. Am J Neuroradiol. 19:1389–1396. 9763366 PMC8338698

[ref7] Baudet M-L , Zivraj KH, Abreu-Goodger C, Muldal A, Armisen J, Blenkiron C, Goldstein LD, Miska EA, Holt CE. 2012. miR-124 acts through CoREST to control onset of Sema3A sensitivity in navigating retinal growth cones. Nat Neurosci. 15:29–38. 10.1038/nn.2979 PMC3661270 22138647

[ref8] Brunstrom JE , Gray-Swain MRR, Osborne PA, Pearlman AL. 1997. Neuronal heterotopias in the developing cerebral cortex produced by neurotrophin-4. Neuron. 18:505–517. 9115743 10.1016/s0896-6273(00)81250-7

[ref9] Chevassus-Au-Louis N , Congar P, Represa A, Ben-Ari Y, Gaïarsa JL. 1998a. Neuronal migration disorders: heterotopic neocortical neurons in CA1 provide a bridge between the hippocampus and the neocortex. Proc Natl Acad Sci USA. 95:10263–10268. 9707635 10.1073/pnas.95.17.10263 PMC21496

[ref10] Chevassus-Au-Louis N , Rafiki A, Jorquera I, Ben-Ari Y, Represa A. 1998b. Neocortex in the hippocampus: an anatomical and functional study of CA1 heterotopias after prenatal treatment with methylazoxymethanol in rats. J Comp Neurol. 394:520–536. 9590559 10.1002/(sici)1096-9861(19980518)394:4<520::aid-cne9>3.0.co;2-3

[ref11] Chmielowska J , Stewart MG, Bourne RC. 1988. Gamma-aminobutyric acid (GABA) immunoreactivity in mouse and rat first somatosensory (SI) cortex: description and comparison. Brain Res. 439:155–168. 3359180 10.1016/0006-8993(88)91472-2

[ref12] Clark MG , Sherman GF, Bimonte HA, Fitch RH. 2000. Perceptual auditory gap detection deficits in male BXSB mice with cerebrocortical ectopias. Neuroreport. 11:693–696. 10757502 10.1097/00001756-200003200-00008

[ref13] De Bernabé DBV , Currier S, Steinbrecher A, Celli J, Van Beusekom E, Van der Zwaag B, Kayserili H, Merlini L, Chitayat D, Dobyns WB, et al. 2002. Mutations in the O-mannosyltransferase gene POMT1 give rise to the severe neuronal migration disorder Walker-Warburg syndrome. Am J Hum Genet. 71:1033–1043. 12369018 10.1086/342975 PMC419999

[ref14] Devisme L , Bouchet C, Gonzalès M, Alanio E, Bazin A, Bessières B, Bigi N, Blanchet P, Bonneau D, Bonnières M, et al. 2012. Cobblestone lissencephaly: neuropathological subtypes and correlations with genes of dystroglycanopathies. Brain. 135:469–482. 22323514 10.1093/brain/awr357

[ref15] Dvořàk K , Feit J, Juránková Z. 1978. Experimentally induced focal microgyria and status verrucosus deformis in rats—pathogenesis and interrelation histological and autoradiographical study. Acta Neuropathol. 44:121–129. 716839 10.1007/BF00691477

[ref16] Dwyer ND , Manning DK, Moran JL, Mudbhary R, Fleming MS, Favero CB, Vock VM, O’Leary DDM, Walsh CA, Beier DR. 2011. A forward genetic screen with a thalamocortical axon reporter mouse yields novel neurodevelopment mutants and a distinct emx2 mutant phenotype. Neural Dev. 6:3. 21214893 10.1186/1749-8104-6-3 PMC3024922

[ref17] Er JC , Leong C, Teoh CL, Yuan Q, Merchant P, Dunn M, Sulzer D, Sames D, Bhinge A, Kim D, et al. 2015. NeuO: a fluorescent chemical probe for live neuron labeling. Angew Chem Int Ed. 54:2442–2446. 10.1002/anie.201408614 25565332

[ref18] Farhy-Tselnicker I , Allen NJ. 2018. Astrocytes, neurons, synapses: a tripartite view on cortical circuit development. Neural Dev. 13:1–12. 29712572 10.1186/s13064-018-0104-y PMC5928581

[ref19] Farrell MA , DeRosa MJ, Curran JG, Lenard Secor D, Cornford ME, Comair YG, Peacock WJ, Shields WD, Vinters H. 1992. Neuropathologic findings in cortical resections (including hemispherectomies) performed for the treatment of intractable childhood epilepsy. Acta Neuropathol. 83:246–259. 1557956 10.1007/BF00296786

[ref20] Ferrer I , Alcántara S, Catalá I, Zújar MJ. 1993. Experimentally induced laminar necrosis, status verrucosus, focal cortical dysplasia reminiscent of microgyria, and porencephaly in the rat. Exp Brain Res. 94:261–269. 8359242 10.1007/BF00230294

[ref21] Frenkel M , Sherman GF, Bashan KA, Galaburda AM, LoTurco JJ. 2000. Neocortical ectopias are associated with attenuated neurophysiological responses to rapidly changing auditory stimuli. Neuroreport. 11:575–579. 10718317 10.1097/00001756-200002280-00029

[ref22] Gabel LA . 2011. Layer I neocortical ectopia: cellular organization and local cortical circuitry. Brain Res. 1381:148–158. 21256119 10.1016/j.brainres.2011.01.040 PMC3082941

[ref23] Gabel LA , LoTurco JJ. 2001. Electrophysiological and morphological characterization of neurons within neocortical ectopias. J Neurophysiol. 85:495–505. 11160488 10.1152/jn.2001.85.2.495

[ref24] Gabel LA , LoTurco JJ. 2002. Layer I ectopias and increased excitability in murine neocortex. J Neurophysiol. 87:2471–2479. 11976384 10.1152/jn.2002.87.5.2471

[ref25] Galaburda AM , Kemper TL. 1979. Cytoarchitectonic abnormalities in developmental dyslexia: a case study. Ann Neurol. 6:94–100. 496415 10.1002/ana.410060203

[ref26] Galaburda AM , Sherman GF, Rosen GD, Aboitiz F, Geschwind N. 1985. Developmental dyslexia: four consecutive patients with cortical anomalies. Ann Neurol. 18:222–233. 4037763 10.1002/ana.410180210

[ref27] Gao PP , Yue Y, Zhang JH, Cerretti DP, Levitt P, Zhou R. 1998. Regulation of thalamic neurite outgrowth by the Eph ligand ephrin-A5: implications in the development of thalamocortical projections. Proc Natl Acad Sci USA. 95:5329–5334. 9560275 10.1073/pnas.95.9.5329 PMC20260

[ref28] Goebbels S , Wieser GL, Pieper A, Spitzer S, Weege B, Yan K, Edgar JM, Yagensky O, Wichert SP, Agarwal A, et al. 2017. A neuronal PI(3,4,5)P3-dependent program of oligodendrocyte precursor recruitment and myelination. Nat Neurosci. 20:10–15. 27775720 10.1038/nn.4425

[ref29] Halfter W , Dong S, Yip Y-P, Willem M, Mayer U. 2002. A critical function of the pial basement membrane in cortical histogenesis. J Neurosci. 22:6029–6040. 12122064 10.1523/JNEUROSCI.22-14-06029.2002 PMC6757907

[ref30] Hardiman O , Burke T, Phillips J, Murphy S, O’Moore B, Staunton H, Farrell MA. 1988. Microdysgenesis in resected temporal neocortex: incidence and clinical significance in focal epilepsy. Neurology. 38:1041–1047. 3386820 10.1212/wnl.38.7.1041

[ref31] Hill RA , Grutzendler J. 2014. *In vivo* imaging of oligodendrocytes with sulforhodamine 101. Nat Methods. 11:1081–1082. 25357236 10.1038/nmeth.3140 PMC4539948

[ref32] Hill RA , Li AM, Grutzendler J. 2018. Lifelong cortical myelin plasticity and age-related degeneration in the live mammalian brain. Nat Neurosci. 21:1–13. 10.1038/s41593-018-0120-6 PMC5920745 29556031

[ref33] Humphreys P , Kaufmann WE, Galaburda AM. 1990. Developmental dyslexia in women: neuropathological findings in three patients. Ann Neurol. 28:727–738. 2285260 10.1002/ana.410280602

[ref34] Hyde LA , Hoplight BJ, Harding S, Sherman GF, Mobraaten LE, Denenberg VH. 2001. Effects of ectopias and their cortical location on several measures of learning in BXSB mice. Dev Psychobiol. 39:286–300. 11745324 10.1002/dev.1006

[ref35] Hyde LA , Sherman GF, Hoplight BJ, Denenberg VH. 2000. Working memory deficits in BXSB mice with neocortical ectopias. Physiol Behav. 70:1–5. 10978470 10.1016/s0031-9384(00)00239-0

[ref36] Ishii K , Kubo KI, Endo T, Yoshida K, Benner S, Ito Y, Aizawa H, Aramaki M, Yamanaka A, Tanaka K, et al. 2015. Neuronal heterotopias affect the activities of distant brain areas and lead to behavioral deficits. J Neurosci. 35:12432–12445. 26354912 10.1523/JNEUROSCI.3648-14.2015 PMC6605399

[ref37] Jacob H . 1940. Die feinere Oberflächengestaltung der Hirnwindungen, die Hirnwarzenbildung und die Mikropolygyrie. Zeitschrift für die gesamte Neurol und Psychiatr. 170:68–84.

[ref38] Jara JH , Gautam M, Kocak N, Xie EF, Mao Q, Bigio EH, Özdinler PH. 2019. MCP1-CCR2 and neuroinflammation in the ALS motor cortex with TDP-43 pathology. J Neuroinflammation. 16:196. 31666087 10.1186/s12974-019-1589-y PMC6822373

[ref39] Jenner AR , Galaburda AM, Sherman GF. 2000. Connectivity of ectopic neurons in the molecular layer of the somatosensory cortex in autoimmune mice. Cerebral Cortex. 10:1005–1013. 11007551 10.1093/cercor/10.10.1005

[ref40] Kakita A , Hayashi S, Moro F, Guerrini R, Ozawa T, Ono K, Kameyama S, Walsh CA, Takahashi H. 2002. Bilateral periventricular nodular heterotopia due to filamin 1 gene mutation: widespread glomeruloid microvascular anomaly and dysplastic cytoarchitecture in the cerebral cortex. Acta Neuropathol. 104:649–657. 12410386 10.1007/s00401-002-0594-9

[ref41] Kasper BS , Stefan H, Buchfelder M, Paulus W. 1999. Temporal lobe microdysgenesis in epilepsy versus control brains. J Neuropathol Exp Neurol. 58:22–28. 10068310 10.1097/00005072-199901000-00003

[ref42] Kaufmann WE , Galaburda AM. 1989. Cerebrocortical microdysgenesis in neurologically normal subjects: a histopathologic study. Neurology. 39:238–244. 2915796 10.1212/wnl.39.2.238

[ref43] Kim J-Y , Ash RT, Ceballos-Diaz C, Levites Y, Golde TE, Smirnakis SM, Jankowsky JL. 2013. Viral transduction of the neonatal brain delivers controllable genetic mosaicism for visualising and manipulating neuronal circuits *in vivo*. Eur J Neurosci. 37:1203–1220. 23347239 10.1111/ejn.12126 PMC3628093

[ref44] Lee KS , Schottler F, Collins JL, Lanzino G, Couture D, Rao A, Hiramatsu KI, Goto Y, Hong SC, Caner H, et al. 1997. A genetic animal model of human neocortical heterotopia associated with seizures. J Neurosci. 17:6236–6242. 9236234 10.1523/JNEUROSCI.17-16-06236.1997 PMC6568362

[ref45] Li X , Cui D, Jiruska P, Fox JE, Yao X, Jefferys JGR. 2007. Synchronization measurement of multiple neuronal populations. J Neurophysiol. 98:3341–3348. 17913983 10.1152/jn.00977.2007

[ref46] Li X , Ouyang G, Usami A, Ikegaya Y, Sik A. 2010. Scale-free topology of the CA3 hippocampal network: a novel method to analyze functional neuronal assemblies. Biophys J. 98:1733–1741. 20441736 10.1016/j.bpj.2010.01.013 PMC2862192

[ref47] Lipoff DM , Bhambri A, Fokas GJ, Sharma S, Gabel LA, Brumberg JC, Richfield EK, Ramos RL, Toia AR, Pasternack DM, et al. 2011. Neocortical molecular layer heterotopia in substrains of C57BL/6 and C57BL/10 mice. Brain Res. 337:36–43. 10.1016/j.brainres.2011.03.026 21419110

[ref48] Lo Turco J , Manent JB, Sidiqi F. 2009. New and improved tools for *in utero* electroporation studies of developing cerebral cortex. Cereb Cortex. 19:i120–i125. 19395528 10.1093/cercor/bhp033 PMC3859267

[ref49] Manent JB , Wang Y, Chang Y, Paramasivam M, LoTurco JJ. 2009. Dcx reexpression reduces subcortical band heterotopia and seizure threshold in an animal model of neuronal migration disorder. Nat Med. 15:84–90. 19098909 10.1038/nm.1897 PMC2715867

[ref50] Meencke HJ , Veith G. 1992. Migration disturbances in epilepsy. Epilepsy Res Suppl. 9:31–39; discussion 39-40. 1285910

[ref51] Métin C , Deléglise D, Serafini T, Kennedy TE, Tessier-Lavigne M. 1997. A role for netrin-1 in the guidance of cortical efferents. Development. 124:5063–5074. 9362464 10.1242/dev.124.24.5063

[ref52] Meyer HS , Schwarz D, Wimmer VC, Schmitt AC, Kerr JND, Sakmann B, Helmstaedter M. 2011. Inhibitory interneurons in a cortical column form hot zones of inhibition in layers 2 and 5A. Proc Natl Acad Sci USA. 108:16807–16812. 21949377 10.1073/pnas.1113648108 PMC3189020

[ref53] Micheva KD , Wolman D, Mensh BD, Pax E, Buchanan J, Smith SJ, Bock DD. 2016. A large fraction of neocortical myelin ensheathes axons of local inhibitory neurons. Elife. 5:e15784. 27383052 10.7554/eLife.15784 PMC4972537

[ref54] Molyneaux BJ , Arlotta P, Fame RM, MacDonald JL, MacQuarrie KL, Macklis JD. 2009. Novel subtype-specific genes identify distinct subpopulations of callosal projection neurons. J Neurosci. 29:12343–12354. 19793993 10.1523/JNEUROSCI.6108-08.2009 PMC2776075

[ref55] Molyneaux BJ , Goff LA, Brettler AC, Chen H-H, Brown JR, Hrvatin S, Rinn JL, Arlotta P. 2015. DeCoN: genome-wide analysis of *in vivo* transcriptional dynamics during pyramidal neuron fate selection in neocortex. Neuron. 85:275–288. 25556833 10.1016/j.neuron.2014.12.024 PMC4430475

[ref56] Moore SA , Saito F, Chen J, Michele DE, Henry MD, Messing A, Cohn RD, Ross-Barta SE, Westra S, Williamson RA, et al. 2002. Deletion of brain dystroglycan recapitulates aspects of congenital muscular dystrophy. Nature. 418:422–425. 12140559 10.1038/nature00838

[ref57] Morel F , Wildi E. 1952. Dysgénésie nodulaire disséminée de l’écorce frontale. Rev Neurol (Paris). 87:251–270. 13014794

[ref58] Myshrall TD , Moore SA, Ostendorf AP, Satz JS, Kowalczyk T, Nguyen H, Daza RAM, Lau C, Campbell KP, Hevner RF. 2012. Dystroglycan on radial glia end feet is required for pial basement membrane integrity and columnar organization of the developing cerebral cortex. J Neuropathol Exp Neurol. 71:1047–1063. 23147502 10.1097/NEN.0b013e318274a128 PMC3512206

[ref59] Nadarajah B , Parnavelas JG. 2002. Modes of neuronal migration in the developing cerebral cortex. Nat Rev Neurosci. 3:423–432. 12042877 10.1038/nrn845

[ref60] Nakano I , Funahashi M, Takada K, Toda T. 1996. Are breaches in the glia limitans the primary cause of the micropolygyria in Fukuyama-type congenital muscular dystrophy (FCMD)? Pathological study of the cerebral cortex of an FCDM fetus. Acta Neuropathol. 91:313–321. 8834545 10.1007/s004010050431

[ref61] Nieto M , Monuki ES, Tang H, Imitola J, Haubst N, Khoury SJ, Cunningham J, Gotz M, Walsh CA. 2004. Expression of Cux-1 and Cux-2 in the subventricular zone and upper layers II-IV of the cerebral cortex. J Comp Neurol. 479:168–180. 15452856 10.1002/cne.20322

[ref62] Nordborg C , Eriksson S, Rydenhag B, Uvebrant P, Malmgren K. 1999. Microdysgenesis in surgical specimens from patients with epilepsy: occurrence and clinical correlations. J Neurol Neurosurg Psychiatry. 67:521–524. 10486403 10.1136/jnnp.67.4.521 PMC1736564

[ref63] Palmini A , Andermann F, Olivier A, Tampieri D, Robitaille Y, Andermann E, Wright G. 1991. Focal neuronal migration disorders and intractable partial epilepsy: a study of 30 patients. Ann Neurol. 30:741–749. 1789691 10.1002/ana.410300602

[ref64] Patel TP , Man K, Firestein BL, Meaney DF. 2015. Automated quantification of neuronal networks and single-cell calcium dynamics using calcium imaging. J Neurosci Methods. 243:26–38. 25629800 10.1016/j.jneumeth.2015.01.020 PMC5553047

[ref65] Peiffer AM , Dunleavy CK, Frenkel M, Gabel LA, LoTurco JJ, Rosen GD, Fitch RH. 2001. Impaired detection of variable duration embedded tones in ectopic NZB/BINJ mice. Neuroreport. 12:2875–2879. 11588594 10.1097/00001756-200109170-00024

[ref66] Phifer CB , Terry LM. 1986. Use of hypothermia for general anesthesia in preweanling rodents. Physiol Behav. 38:887–890. 3823208 10.1016/0031-9384(86)90058-2

[ref67] Polleux F , Giger RJ, Ginty DD, Kolodkin AL, Ghosh A. 1998. Patterning of cortical efferent projections by semaphorin-neuropilin interactions. Science. 282:1904–1906. 9836643 10.1126/science.282.5395.1904

[ref68] Radner S , Banos C, Bachay G, Li YN, Hunter DD, Brunken WJ, Yee KT. 2013. β2 and γ3 laminins are critical cortical basement membrane components: ablation of Lamb2 and Lamc3 genes disrupts cortical lamination and produces dysplasia. Dev Neurobiol. 73:209–229. 22961762 10.1002/dneu.22057

[ref69] Raedler E , Raedler A. 1978. Autoradiographic study of early neurogenesis in rat neocortex. Anat Embryol. 154:267–284. 10.1007/BF00345657 707818

[ref70] Rakic P . 1972. Mode of cell migration to the superficial layers of fetal monkey neocortex. J Comp Neurol. 145:61–83. 4624784 10.1002/cne.901450105

[ref71] Ramos RL , Siu NY, Brunken WJ, Yee KT, Gabel LA, Van Dine SE, Hoplight BJ. 2014. Cellular and axonal constituents of neocortical molecular layer heterotopia. Dev Neurosci. 36:477–489. 25247689 10.1159/000365100

[ref72] Ramos RL , Smith PT, DeCola C, Tam D, Corzo O, Brumberg JC. 2008. Cytoarchitecture and transcriptional profiles of neocortical malformations in inbred mice. Cereb Cortex. 18:2614–2628. 18308707 10.1093/cercor/bhn019 PMC2733319

[ref73] Richards LJ , Koester SE, Tuttle R, O’Leary DD. 1997. Directed growth of early cortical axons is influenced by a chemoattractant released from an intermediate target. J Neurosci. 17:2445–2458. 9065505 10.1523/JNEUROSCI.17-07-02445.1997 PMC6573499

[ref74] Riggs HE , McGrath JJ, Schwarz HP. 1956. Malformation of the adult brain (albino rat) resulting from prenatal irradiation. J Neuropathol Exp Neurol. 15:432–447. 13367864 10.1097/00005072-195610000-00006

[ref75] Romero DM , Bahi-buisson N, Francis F. 2018. Genetics and mechanisms leading to human cortical malformations. Semin Cell Dev Biol. 76:33–75. 28951247 10.1016/j.semcdb.2017.09.031

[ref76] Roper SN , Gilmore RL, Houser CR. 1995. Experimentally induced disorders of neuronal migration produce an increased propensity for electrographic seizures in rats. Epilepsy Res. 21:205–219. 8536674 10.1016/0920-1211(95)00027-8

[ref77] Rosen GD , Bai J, Wang Y, Fiondella CG, Threlkeld SW, LoTurco JJ, Galaburda AM. 2007. Disruption of neuronal migration by RNAi of Dyx1c1 results in neocortical and hippocampal malformations. Cereb Cortex. 17:2562–2572. 17218481 10.1093/cercor/bhl162 PMC3742088

[ref78] Rosen GD , Sherman GF, Richman JM, Stone LV, Galaburda AM. 1992. Induction of molecular layer ectopias by puncture wounds in newborn rats and mice. Dev Brain Res. 67:285–291. 1380903 10.1016/0165-3806(92)90229-p

[ref79] Saito T . 2006. *In vivo* electroporation in the embryonic mouse central nervous system. Nat Protoc. 1:1552–1558. 17406448 10.1038/nprot.2006.276

[ref80] Saito T , Nakatsuji N. 2001. Efficient gene transfer into the embryonic mouse brain using *in vivo* electroporation. Dev Biol. 240:237–246. 11784059 10.1006/dbio.2001.0439

[ref81] Schain AJ , Hill RA, Grutzendler J. 2014. Label-free *in vivo* imaging of myelinated axons in health and disease with spectral confocal reflectance microscopy. Nat Med. 20:443–449. 24681598 10.1038/nm.3495 PMC3981936

[ref82] Schottler F , Fabiato H, Leland JM, Chang LY, Lotfi P, Getachew F, Lee KS. 2001. Normotopic and heterotopic cortical representations of mystacial vibrissae in rats with subcortical band heterotopia. Neuroscience. 108:217–235. 11734356 10.1016/s0306-4522(01)00395-5

[ref83] Schulze KD , Braak H. 1978. Hirnwarzen. Zeitschrift fur Mikroskopisch-Anatomische Forsch Abteilung 2. 92:609–623. 749392

[ref84] Sherman GF , Galaburda AM, Behan PO, Rosen GD. 1987. Neuroanatomical anomalies in autoimmune mice. Acta Neuropathol. 74:239–242. 3673516 10.1007/BF00688187

[ref85] Sherman GF , Galaburda AM, Geschwind N. 1985. Cortical anomalies in brains of New Zealand mice: a neuropathologic model of dyslexia? Proc Natl Acad Sci USA. 82:8072–8074. 3865217 10.1073/pnas.82.23.8072 PMC391444

[ref86] Sherman GF , Rosen GD, Stone LV, Press DM, Galaburda AM. 1992. The organization of radial glial fibers in spontaneous neocortical ectopias of newborn New Zealand black mice. Dev Brain Res. 67:279–283. 1511521 10.1016/0165-3806(92)90228-o

[ref87] Sherman GF , Stone JS, Press DM, Rosen GD, Galaburda AM. 1990. Abnormal architecture and connections disclosed by neurofilament staining in the cerebral cortex of autoimmune mice. Brain Res. 529:202–207. 2126480 10.1016/0006-8993(90)90828-y

[ref88] Shewan D , Dwivedy A, Anderson R, Holt CE. 2002. Age-related changes underlie switch in netrin-1 responsiveness as growth cones advance along visual pathway. Nat Neurosci. 5:955–962. 12352982 10.1038/nn919

[ref89] Singh SC . 1977. Ectopic neurones in the hippocampus of the postnatal rat exposed to methylazoxymethanol during foetal development. Acta Neuropathol. 40:111–116. 930560 10.1007/BF00688698

[ref90] Sisodiya SM . 2004. Malformations of cortical development: burdens and insights from important causes of human epilepsy. Lancet Neurol. 3:29–38. 14693109 10.1016/s1474-4422(03)00620-3

[ref91] Skaliora I , Singer W, Betz H, Püschel AW. 1998. Differential patterns of semaphorin expression in the developing rat brain. Eur J Neurosci. 10:1215–1229. 9749776 10.1046/j.1460-9568.1998.00128.x

[ref92] Sorensen SA , Bernard A, Menon V, Royall JJ, Glattfelder KJ, Desta T, Hirokawa K, Mortrud M, Miller JA, Zeng H, et al. 2015. Correlated gene expression and target specificity demonstrate excitatory projection neuron diversity. Cereb Cortex. 25:433–449. 24014670 10.1093/cercor/bht243

[ref93] Sturrock RR . 1980. Myelination of the mouse corpus callosum. Neuropathol Appl Neurobiol. 6:415–420. 7453945 10.1111/j.1365-2990.1980.tb00219.x

[ref94] Tabata H , Nakajima K. 2001. Efficient *in utero* gene transfer system to the developing mouse brain using electroporation: visualization of neuronal migration in the developing cortex. Neuroscience. 103:865–872. 11301197 10.1016/s0306-4522(01)00016-1

[ref95] Toia AR , Cuoco JA, Esposito AW, Ahsan J, Joshi A, Herron BJ, Torres G, Bolivar VJ, Ramos RL. 2017. Divergence and inheritance of neocortical heterotopia in inbred and genetically-engineered mice. Neurosci Lett. 638:175–180. 27993709 10.1016/j.neulet.2016.12.038 PMC5239770

[ref96] Tomassy GS , Berger DR, Chen HH, Kasthuri N, Hayworth KJ, Vercelli A, Seung HS, Lichtman JW, Arlotta P. 2014. Distinct profiles of myelin distribution along single axons of pyramidal neurons in the neocortex. Science. 344:319–324. 24744380 10.1126/science.1249766 PMC4122120

[ref97] Wang B , Ji L, Bishayee K, Li C, Huh SO. 2020. Identification and prevention of heterotopias in mouse neocortical neural cell migration incurred by surgical damages during utero electroporation procedures. Animal Cells Syst. 24:114–123. 10.1080/19768354.2020.1737225 PMC7241496 32489691

[ref98] Waters NS , Sherman GF, Galaburda AM, Denenberg VH. 1997. Effects of cortical ectopias on spatial delayed-matching-to-sample performance in BXSB mice. Behav Brain Res. 84:23–29. 9079769 10.1016/s0166-4328(96)00130-1

[ref99] Yamamoto T , Toyoda C, Kobayashi M, Kondo E, Saito K, Osawa M. 1997. Pial-glial barrier abnormalities in fetuses with Fukuyama congenital muscular dystrophy. Brain Dev. 19:35–42. 9071488 10.1016/s0387-7604(96)00056-3

