# Supplementary material for: Intravital Imaging of Neocortical Heterotopia Reveals Aberrant Axonal Pathfinding and Myelination around Ectopic Neurons
Source: Cereb Cortex. 2021 Apr 20;31(9):4340–56. doi: 10.1093/cercor/bhab090 (PMC8328209; doi:10.1093/cercor/bhab090)
Supplement: Supplementary_Data_final_bhab090 [file supplementary_data_final_bhab090.docx]

**SUPPLEMENTARY DATA**

**Manuscript Title**

Intravital imaging of neocortical heterotopia reveals aberrant axonal pathfinding and myelination around ectopic neurons

**Authors**

Alice M. Li, Robert A. Hill*, and Jaime Grutzendler*

*Correspondence

Robert Hill: [robert.hill@dartmouth.edu](mailto:robert.hill@dartmouth.edu)

Jaime Grutzendler: [jaime.grutzendler@yale.edu](mailto:jaime.grutzendler@yale.edu)

**This PDF file includes**

Supplementary Figures 1-4

Supplementary Table 1

**
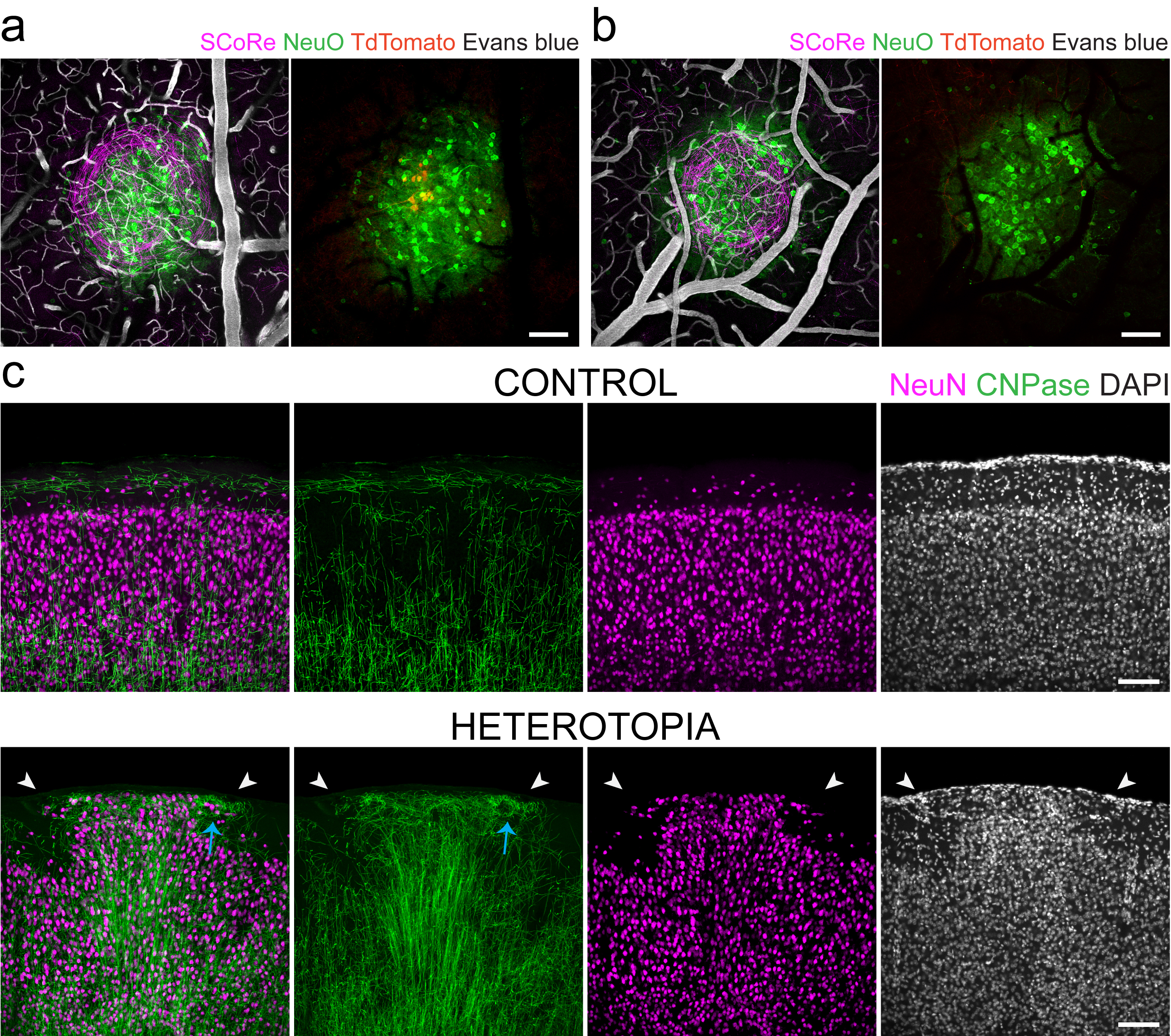
**

**Supplementary Figure 1. Myelinated axons project aberrantly around heterotopic cells. a-b,** Low magnification *in vivo* images captured from layer I of the mouse cortex showing the pronounced swirling of myelinated fibers around heterotopic neuron cell bodies (NeuO, green) including those co-labelled with TdTomato fluorescent protein (red) by IUE. Images in (a) are of the same heterotopion shown in Figures 1c and 2a. Images in (b) are of the same heterotopion shown in Figure 4a (middle column). Scale bars, 100 µm **c**, High resolution immunostaining showing the winding trajectories followed by CNPase^+^ fibers (bottom, blue arrow) within a layer I heterotopion (NeuN; white arrowheads) in a P30 mouse coronal section. The aberrantly-projecting myelinated fiber bundles are absent from immediately adjacent non-heterotopic cortex and corresponding contralateral control regions (top row). Images are representative of experiments performed in at least three animals. Scale bars, 100 µm.

**
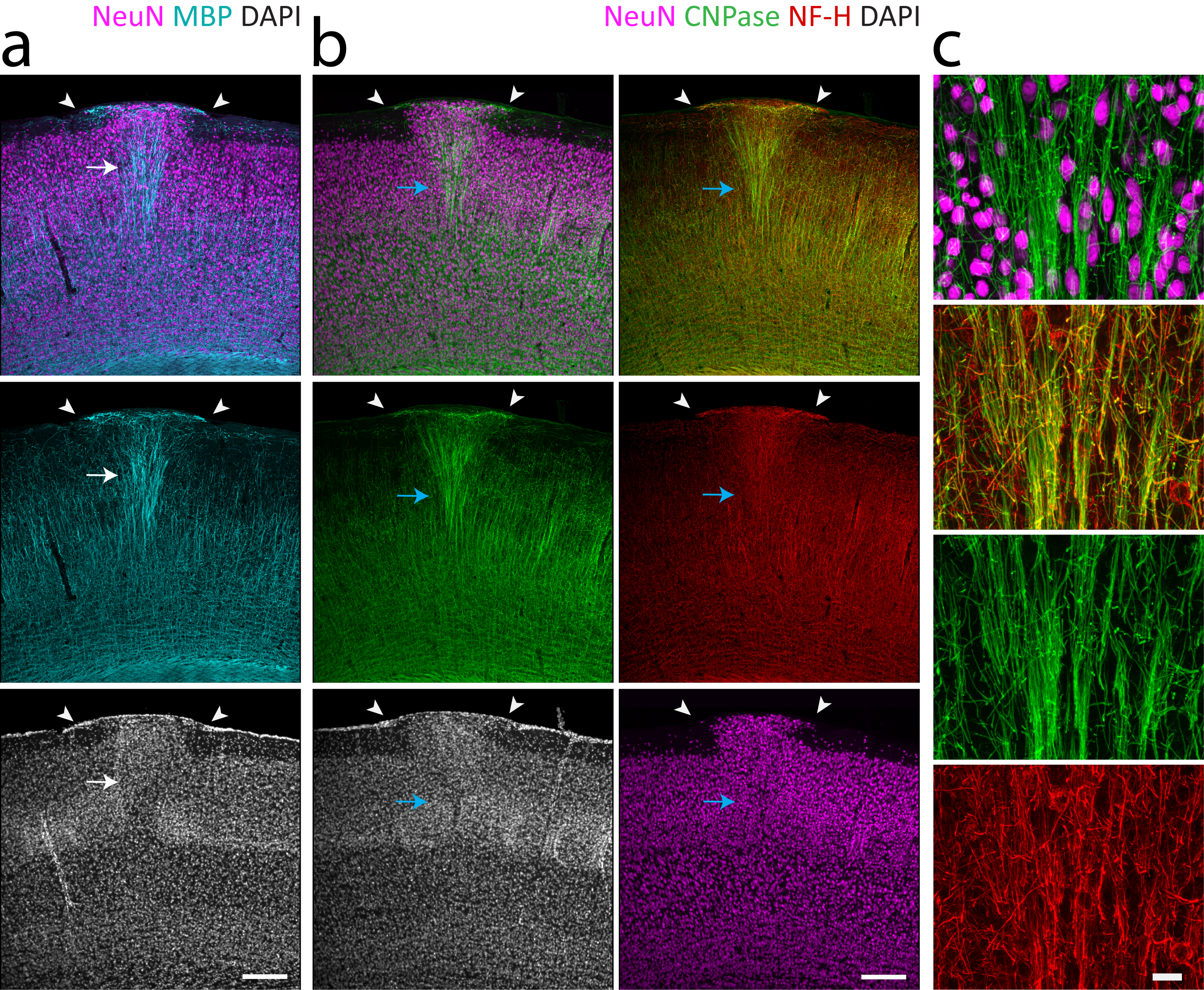
**

**Supplementary Figure 2. Disrupted myelin patterning and axonal pathfinding occurs in deeper cortical layers. a,** Myelinated fibers (MBP, cyan) accumulate in thick radially oriented cords (white arrow) beneath a layer I heterotopion (NeuN; arrowheads) in an immunostained P30 mouse forebrain coronal section. **b-c,**Confocal images of a different coronal section from the same heterotopion in (**a**), revealing similar aberrant myelin and axon accumulations as identified by oligodendrocyte CNPase (green) and axonal NF-H (red) immunostaining, respectively**.**A higher magnification view of the densely packed myelinated axons fasciculating underneath the heterotopion (**b**, blue arrow) is shown in (**c**). Images in (**b**) and (**c**) are of same heterotopion shown in Figure 3e**.** Images are representative of fixed tissue experiments performed in at least three mice. Scale bars, 200 µm (**a**, **b**), and 20 µm (**c**).

**
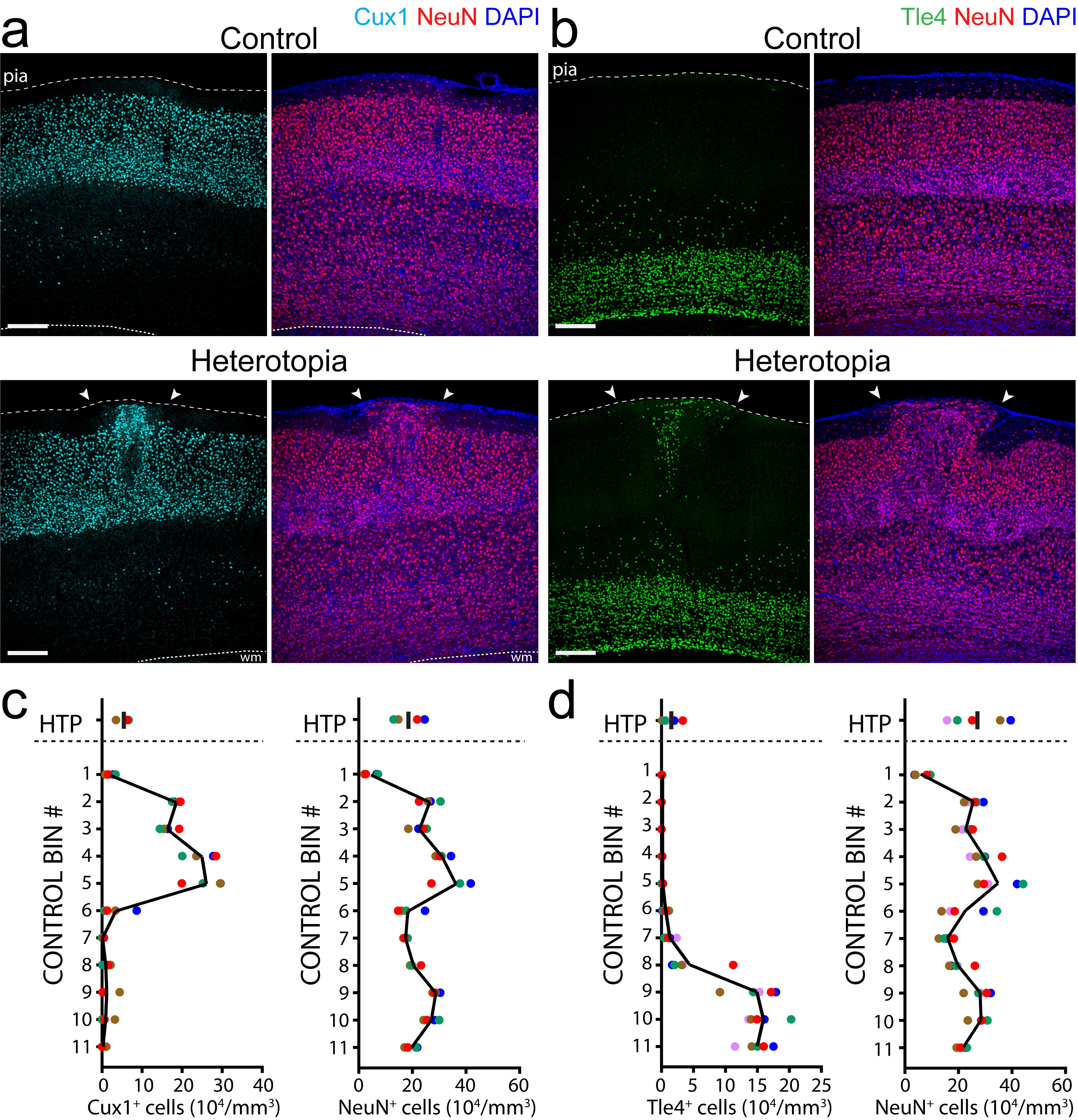
**

**Supplementary Figure 3. Heterotopic neurons express both deep and superficial cortical layer markers. a**-**b**, Additional fixed tissue images captured from the heterotopia displayed in Figures 7b and c, confirming the presence of Cux1^+^ (**a**, cyan) and Tle4^+^ (**b**, green) neuronal cell bodies within the representative heterotopia (bottom images, arrowheads). In corresponding contralateral control regions (top images), Cux1 (left) largely concentrates in superficial cortical layers whereas Tle4 (right) tends to localize toward deeper cortical layers. **c**-**d**, Quantifications showing the densities of Cux1^+^ (**c**, *n* = 4 animals), Tle4^+^ (**d**, *n* = 5 animals), and NeuN^+^ (**c** and **d**, right) cells in heterotopia and across corresponding contralateral control regions of P30 mice. Each dot corresponds to the layer I heterotopion or control bin of a single animal, with all dots of the same color belonging to the same animal. The black line denotes the mean. Images are representative of experiments performed in at least four animals. WM, white matter; HTP, heterotopia. Scale bars, 200 µm.

**
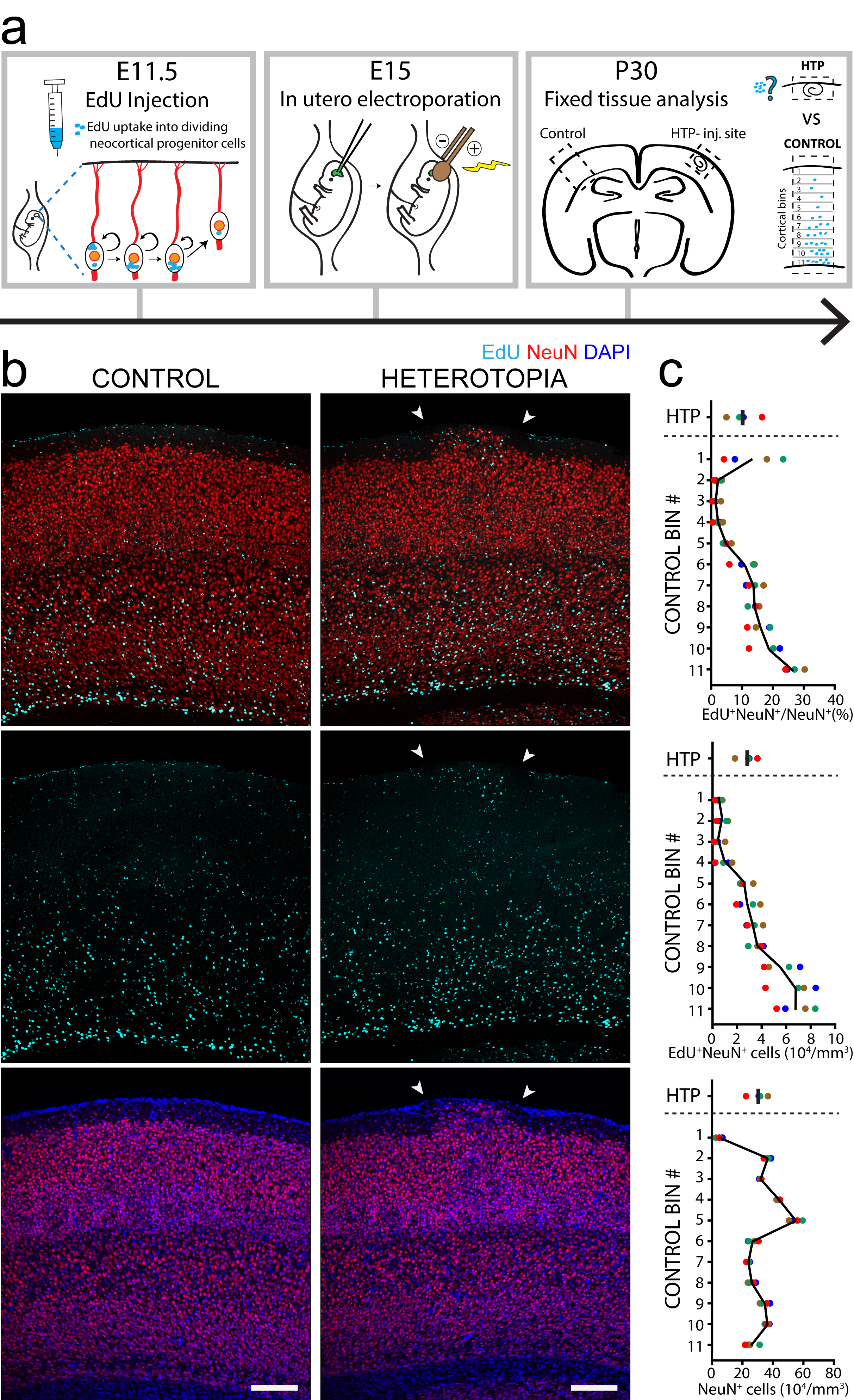
Supplementary Figure 4. Birth-dating of heterotopic neurons. a**, Diagram of the pulse labelling strategy for birthdating heterotopic cortical neurons. Dividing cells are labelled by a single EdU injection at E11.5, with heterotopia induced at E15. Mice are sacrificed at P30 for analysis. **b**, Low magnification images showing the EdU^+^ neurons incorporated in a layer I heterotopion (right, arrowheads) of a P30 mouse. In corresponding contralateral control cortex (left column), EdU expression is predominantly concentrated in deeper cortical layers. **c**, Quantifications showing the percentages of NeuN^+^ cells that also express EdU (top), EdU^+^NeuN^+^ cell body densities (middle), and NeuN^+^ cell body densities (bottom) in layer I heterotopia and across corresponding contralateral control cortices of P30 mice. Each dot corresponds to the layer I heterotopion or control bin of a single animal, with all dots of the same color belonging to the same animal (*n* = 4 animals total). Images are representative of the observations made in each mouse brain. The black line denotes the mean. WM, white matter; HTP, heterotopia. Scale bars, 200 µm.

| **Figure Number** | **Statistical Test** | **Sample Size** | **Sample Definition** | **P Value** | **Degrees of Freedom and F/T/z/R/ETC value** |
| --- | --- | --- | --- | --- | --- |
| 1b | Wilcoxon non-parametric matched-pairs signed rank test | 6 | number of mice (paired samples) | p = 0.0313 for NeuO, HTP vs. Control | n/a |
| 2d | Wilcoxon non-parametric matched-pairs signed rank test | 8 | number of mice (paired samples) | SCoRe density,  p=0.0078 for HTP edge vs. Control; p=0.0078 for HTP center vs. Control | n/a |
|  | Wilcoxon non-parametric matched-pairs signed rank test | 6 | number of mice (paired samples) | p = 0.0313 for CNPase density, HTP vs. Control. | n/a |
|  | Wilcoxon non-parametric matched-pairs signed rank test | 6 | number of mice (paired samples) | p = 0.0313 for CNPase^+^ cell bodies, HTP vs. Control. | n/a |
| 5c | Wilcoxon non-parametric matched-pairs signed rank test | 6 | number of mice (paired samples) | p > 0.9999 for CNPase^+^ cell bodies, HTP vs. Control. | n/a |
|  | Wilcoxon non-parametric matched-pairs signed rank test | 6 | number of mice (paired samples) | p = 0.0625 for CNPase density, HTP vs. Control. | n/a |
| 6c | Wilcoxon non-parametric matched-pairs signed rank test | 6 | number of mice (paired samples) | p = 0.8438 for Aldh1L1^+^ cell density, HTP vs. Control. | n/a |
| 6f | Wilcoxon non-parametric matched-pairs signed rank test | 6 | number of mice (paired samples) | p = 0.9375 for Iba^+^ cell density, HTP vs. Control | n/a |
| 7f | Wilcoxon non-parametric matched-pairs signed rank test | 6 | number of mice (paired samples) | p = 0.5313 for GAD67^+^ cell density, HTP vs Control | n/a |
| 8d | Wilcoxon non-parametric matched-pairs signed rank test | 7 | number of mice (paired samples) | p = 0.6875 for spike event frequency, HTP vs Layers II/III | n/a |
|  | Wilcoxon non-parametric matched-pairs signed rank test | 7 | number of mice (paired samples) | p = 0.9375 for variance (s.d.), HTP vs. Layers II/III | n/a |
|  | Wilcoxon non-parametric matched-pairs signed rank test | 7 | number of mice (paired samples) | p = 0.2969 for global synchronization index, HTP vs. Layers II/III | n/a |

**Supplementary Table 1.** Details for all statistical analyses, including sample sizes and p-values.
