# Supplementary material for: Intravital Imaging of Neocortical Heterotopia Reveals Aberrant Axonal Pathfinding and Myelination around Ectopic Neurons
Source: Cereb Cortex. 2021 Apr 20;31(9):4340–56. doi: 10.1093/cercor/bhab090 (PMC8328209; doi:10.1093/cercor/bhab090)
Supplement: Supp_Video_Legends_bhab090 [file supp_video_legends_bhab090.docx]

**Video 1. *In vivo* imaging of a layer I heterotopion and adjacent control area.** Confocal z-stacks of a heterotopion (right) and ipsilateral non-injected layer I control region (left) captured from a living P30 mouse using label-free SCoRe (magenta) and fluorescence microscopy, showing the aberrantly projecting myelinated axons and ectopic neurons located inside a layer I heterotopion induced at E15. Neuronal cell bodies and cerebral blood vessels are visualized using fluorescent neuronal dye NeuO (green) and Evans Blue (white), respectively. The density of the horizontally crisscrossing, aberrantly projecting fibers diminishes with increasing cortical depth (indicated at bottom left).

**Video 2. Layer I heterotopia display aberrant axon pathfinding and exuberant myelination *in vivo.*** Three-dimensional (3D) z-stacks captured from the heterotopia displayed in Supplementary Figure 1a (left) and Supplementary Figure 1b (right), confirming the pronounced changes in axon pathfinding and myelination around layer I heterotopic neuron cell bodies (NeuO; green), as revealed by SCoRe microscopy (magenta) and confocal fluorescence imaging *in vivo*. Some neuronal cell bodies display evidence of colabelling (TdTomato; red) by E15 IUE-mediated transfection. Images are representative of experiments performed in at least three animals.

**Video 3. Layer I heterotopia and adjacent layer II/III regions display highly variable spontaneous neuronal calcium transients.** Representative *in vivo* two-photon time-lapse recordings of GCaMP6f-labelled neurons (green) within a layer I heterotopion (right) and an adjacent layer II/III cortical region (left) in an awake, head-fixed P55 mouse. Diverse patterns of spontaneous calcium transients are observed in neuronal cell bodies throughout both imaged regions. Note the absence of obvious epileptiform activity. Images were acquired at 2 Hz from an E15-induced layer I heterotopion. Some neuronal cell body and axonal labelling by pCAG-TdTomato (red) is the result of E15 IUE-mediated transfection.

**Video 4. Heterotopic neurons respond to sensory stimuli.** Two *in vivo* confocal time-lapse recording examples of GCaMP6f-labelled neurons within a layer I heterotopion before, during, and after the application of a brief whisker stimulus, administered one minute into each imaging session. The timing of the stimulus and accompanying movements are indicated by the white circle (positioned at the top left of the embedded video). Neuronal calcium changes are observed upon the application of each stimulus, indicating the responsiveness of the heterotopic neurons to sensorimotor input. Both recordings were obtained from P55 awake, head-fixed mice with layer I heterotopia that were induced at E15 and virally transfected using AAV-GCaMP6f at P21-P30.
